# Genetically Predicted Homocysteine Levels and B Vitamins on Sarcopenia‐Related Traits: Insights From an Observational and Mendelian Randomization Analysis

**DOI:** 10.1002/fsn3.71556

**Published:** 2026-02-15

**Authors:** Caizheng Yang, Shanshan Ge, Fangying Tian, Yan Jiang, Yue Guo, Xiumei Wang, Hongwei Wang

**Affiliations:** ^1^ Department of Nursing Shanxi Technology and Business University Taiyuan China; ^2^ Department of Health Management The First Hospital of Shanxi Medical University Taiyuan China; ^3^ Department of Hospital‐Acquired Infection Management The Second Hospital of Shanxi Medical University Taiyuan China; ^4^ Department of Nursing, Shanxi Bethune Hospital Shanxi Academy of Medical Sciences, Third Hospital of Shanxi Medical University, Tongji Shanxi Hospital Taiyuan China; ^5^ Department of Neurosurgery, Shanxi Provincial People's Hospital The Fifth Clinical Medical College of Shanxi Medical University Taiyuan China

**Keywords:** B vitamins, homocysteine, mendelian randomization, observational study, sarcopenia

## Abstract

Sarcopenia is a significant public health concern that adversely affects the health and quality of life of older adults. The causal and longitudinal relationships between homocysteine (Hcy), B vitamins, and sarcopenia remain unclear. This study integrated genetic evidence with clinical cohort data to investigate these associations using a two‐stage design. First, we performed a two‐sample Mendelian randomization (MR) analysis using summary data from large‐scale genome‐wide association studies (GWAS) of European ancestry. We examined the potential causal effects of Hcy, Vit B_6_, folate, and Vit B_12_ on sarcopenia‐related phenotypes, including appendicular lean mass (ALM), grip strength, and walking pace, using the inverse‐variance weighted (IVW) method as the primary analysis. Second, to validate these genetic findings and examine their longitudinal relevance, we established an independent retrospective clinical cohort of 1322 individuals. Group‐based trajectory modeling identified distinct Hcy trajectory groups, and multivariable Cox regression with restricted cubic splines was used to assess longitudinal associations and dose–response relationships with incident sarcopenia. The MR analysis showed that genetically predicted higher Hcy levels were causally associated with low grip strength (OR = 1.133, 95% CI: 1.016–1.263, *p* = 0.025) and lower ALM (*β* = −0.043, 95% CI: −0.069 – −0.016, *p* = 0.001). In the clinical cohort, individuals in the medium‐stable and high‐stable Hcy trajectory groups had a 1.965‐fold (95% CI: 1.027–3.759) and 2.832‐fold (95% CI: 1.608–4.987) higher risk of developing sarcopenia, respectively, compared to the low‐stable group. A continuous, incremental dose–response relationship was observed between baseline Hcy levels and sarcopenia risk (*p* < 0.05). No robust genetic evidence supported causal roles for B vitamins in sarcopenia. This study provides evidence that Hcy is associated with sarcopenia risk, suggesting that interventions targeting Hcy may help prevent or delay sarcopenia onset.

## Graphical abstract

We employed Mendelian randomization analysis and longitudinal cohort data to identify a causal relationship between genetically predicted high Hcy levels and low grip strength as well as low appendicular lean mass. In the clinical cohort, individuals with persistent medium and high levels of Hcy had a significantly increased risk of sarcopenia, and a dose–response relationship was observed. No consistent causal evidence was found between B vitamins and the characteristics of sarcopenia.

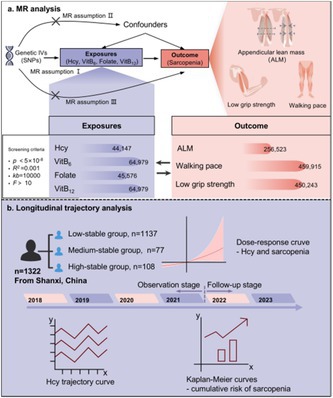

## 1 Background

Sarcopenia is a multigenic complex disease characterized by a progressive decline in muscle mass and function with age, which is one of the main causes of adverse outcomes such as falls, fractures, infections, metabolic disorders, disability, or death in older adults (Cruz‐Jentoft and Sayer 2019; Cruz‐Jentoft et al. 2019; Gielen et al. 2023; Papadopoulou 2020). A meta‐analysis of 263 studies showed that the prevalence of sarcopenia was 8%–36% in individuals < 60 years of age and 10%–27% in those ≥ 60 years of age globally (Petermann‐Rocha et al. 2022). Furthermore, the prevalence of sarcopenia increases with age, and muscle mass and function decline gradually after age 40, with a faster decline after age 60 (Cruz‐Jentoft and Sayer 2019; Cruz‐Jentoft et al. 2019). Studies have shown that sarcopenia is prevalent in patients with chronic kidney disease (especially those receiving dialysis) and is associated with higher mortality rates (Duarte et al. 2024; Shu et al. 2022). In summary, tracking and studying the progression of sarcopenia is crucial to minimizing its threat to the health of the elderly.

The pathogenesis of sarcopenia is multifactorial. The Asian Working Group on Sarcopenia (AWGS) reported in 2019 that the occurrence of sarcopenia is related to aging, cell apoptosis, motor neuron loss, mitochondrial dysfunction, nutritional/absorption disorders, and inflammatory responses. Among them, nutrient intake insufficiency or absorption disorders are important factors in the occurrence of sarcopenia (Chen et al. 2020; Hanach et al. 2019). Older adults are at a notably higher risk of insufficient intake or impaired absorption of several micronutrients critical for muscle homeostasis. This is attributable to age‐related factors such as reduced dietary variety, decreased appetite, altered gastrointestinal function, and increased prevalence of chronic conditions affecting nutrient utilization (Cai, Gao, et al. 2025; Cai, Man, et al. 2025). Of particular relevance are the B vitamins involved in homocysteine (Hcy) metabolism—folate, Vit B_6_, and Vit B_12_—whose suboptimal status is frequently observed in the elderly population (Zhao et al. 2024; Yang et al. 2024; He and Li 2023). Biochemically, Hcy is metabolized via two major pathways: remethylation and transsulfuration. In the remethylation pathway, folate‐derived 5‐methyltetrahydrofolate serves as a methyl donor, and methionine synthase—dependent on Vit B_12_ as a cofactor—converts Hcy to methionine. Alternatively, in the transsulfuration pathway, cystathionine *β*‐synthase, which requires Vit B_6_ as a coenzyme, catalyzes the conversion of Hcy to cystathionine. Consequently, deficiency in any of these B vitamins impairs Hcy clearance, leading to its accumulation in the circulation (Jakubowski 2019; Thomas‐Valdés et al. 2017). This intimate metabol observational efforts to disentangle the individual contributions of Hcy and B vitamins to sarcopenia risk. Prior research has indicated an association between elevated Hcy levels, inadequate B vitamins intake, and sarcopenia (Ter Borg et al. 2016; Kato et al. 2024). However, conflicting results have also been reported (Choi et al. 2023). These conflicting findings highlight a significant gap in knowledge: whether these associations are causal or merely reflective of confounding factors and reverse causality inherent in observational designs.

Among various methods for correlative research, randomized controlled trials (RCTs) provide higher level evidence but are more challenging to implement. Observational studies are influenced by confounding factors and reverse causation, limiting the inference of causal relationships between exposure factors (or intervention measures) and disease outcomes. Mendelian randomization (MR), utilizing single nucleotide polymorphisms (SNPs) as instrumental variables (IVs), is a causal inference method based on genetic variation that offers an effective approach to address these issues (Larsson et al. 2023; Sekula et al. 2016; Cai, Gao, et al. 2025; Cai, Man, et al. 2025). The MR method has been applied in sarcopenia research to explore the causal roles of various risk factors. For instance, previous studies have verified the significant causal effects of allergic phenotypes (such as atopic dermatitis, asthma, eosinophil count) on the pre‐sarcopenia stage and limb lean body mass, and have evaluated the potential impact of metformin‐related drug targets (such as GDF15) on the characteristics of sarcopenia (Tang et al. 2023; Hu et al. 2025; Qi et al. 2025). However, MR estimates reflect lifelong genetic effects and do not directly capture the dynamic progression of biomarkers in clinical populations. To bridge this gap and translate our genetic findings into clinical insight, we employed a two‐stage design that integrates MR with an independent longitudinal cohort. This approach allows us to trace the longitudinal trajectory of Hcy in a clinical setting while establishing the temporal and dose–response relationships with sarcopenia risk, thereby providing robust clinical validation for Hcy and B vitamins as potential targets for nutritional prevention.

## 2 Methods

### 2.1 Study Design

**FIGURE 1:**
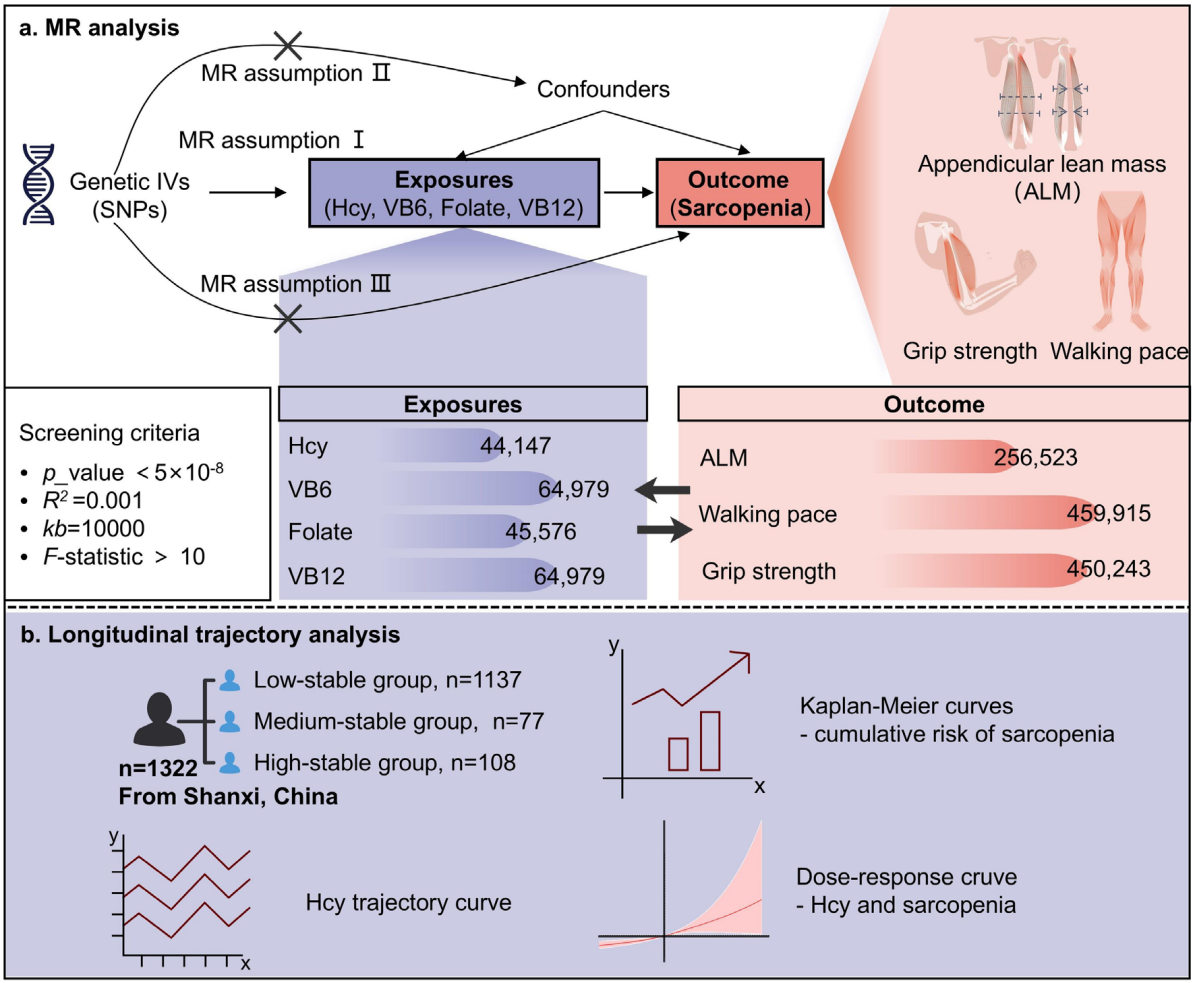
Study design and analytical framework for this study. (a) Schematic diagram of the MR analysis. Genetic instruments (single nucleotide polymorphisms, SNPs) for exposures (homocysteine [Hcy] and B vitamins) were selected based on genome‐wide significance (*p* < 5 × 10^−8^), linkage disequilibrium (*R* ^2^ < 0.001, kb = 10,000), and instrument strength (*F*‐statistic > 10). The diagram illustrates the three key MR assumptions. Sample sizes for each exposure and outcome (sarcopenia‐related traits) are indicated. (b) Longitudinal trajectory analysis and dose–response relationship. Three distinct Hcy trajectory groups (low‐stable, medium‐stable, high‐stable) were identified in a cohort of 1322 individuals from Shanxi, China. Associated Kaplan–Meier curves for sarcopenia risk and the dose–response relationship between Hcy levels and sarcopenia.

We used GWAS summary‐level data and conducted a two‐sample MR analysis to explore the genetic impact of Hcy and B vitamins on sarcopenia‐related traits. To accurately establish causal effects, MR analysis relies on three key assumptions: (1) the association assumption, which requires highly correlated instrumental variables (IVs) with our target exposure factors; (2) the independence assumption, which necessitates that the included IVs are unrelated to confounding factors associated with the exposure‐outcome relationship; and (3) the exclusion restriction assumption, which stipulates that IVs only affect outcomes through their influence on exposure factors, thereby precluding level pleiotropy. To enhance precision and standardization in our research, we followed the guidelines outlined in Strengthening the Reporting of Observational Studies in Epidemiology using Mendelian Randomization (STRBOE‐MR) when reporting our findings (Skrivankova et al. 2021), as presented in the Table S1. Figure 1a illustrates the design flowchart for our MR study.

In addition, we conducted a retrospective longitudinal cohort study to complement the genetic evidence with clinical observations. The study population comprised 1322 individuals who underwent annual health examinations at the Health Management Center of the First Hospital of Shanxi Medical University between January 2018 and December 2021. These participants were prospectively followed from January 2022 to December 2023 to monitor incident cases of sarcopenia. Eligibility criteria were systematically applied during participant selection. Participants were included if they met the following criteria: (1) age 18 years or older; (2) permanent residency in Shanxi Province; (3) availability of complete baseline information; and (4) completion of annual health check‐ups with comprehensive data from 2018 to 2021 without sarcopenia diagnosis. Exclusion criteria: (1) preexisting clinical diagnosis of sarcopenia or secondary sarcopenia due to chronic conditions (including osteoporosis, diabetes, cardiovascular diseases, hepatic disorders, and renal diseases); (2) missing key observational data during follow‐up; (3) administration of Hcy‐lowering or sarcopenia‐specific interventions during the study period; and (4) incomplete baseline demographic information. The overall design of the cohort study is presented in Figure 1b.

### 2.2 Data Sources

In the MR analysis, The definition of sarcopenia followed the criteria established by the European Working Group on Sarcopenia in Older People (EWGSOP) in 2019 (Cruz‐Jentoft and Sayer 2019; Cruz‐Jentoft et al. 2019). We selected low grip strength and walking pace as measures of muscle function and ALM as a measure of muscle mass, which is considered a key component of sarcopenia. Additionally, in order to minimize racial heterogeneity, our analysis will be restricted to individuals of European descent. We followed the definition of hand grip strength proposed by EWGSOP in 2010 (hand grip strength < 30 kg for males; < 20 kg for females), and screened genetic tools from the current largest meta‐analysis. This meta‐analysis included 256,523 individuals of European ancestry aged 60 or above. Among them, 46,596 (18.9%) had muscle weakness based on hand grip strength (Jones et al. 2021). Moreover, the genetic tools for ALM (*n* = 450,243) and Walking pace (*n* = 459,915) were derived from GWAS studies conducted in the UK Biobank. For the study on ALM, participants aged between 48 and 73 from various locations in the UK were recruited. The sum of fat‐free mass of the arms and legs was measured through bioelectrical impedance analysis (BIA). A total of 1059 conditionally independent variations accounted for approximately 15.5% of the phenotypic variance of ALM (Ye et al. 2023). Second, the study on genetic tools for Walking pace originated from a GWAS study with 9,851,867 SNP quantities in the UK Biobank. The researchers captured data on walking pace through touchscreen questionnaires (https://gwas.mrcieu.ac.uk/datasets/ukb‐b‐4711/, accessed July 15th, 2024). The exposure data for Hcy (*n* = 44,147) (van Meurs et al. 2013), Vit B_6_ (*n* = 64,979), Vit B_12_ (*n* = 45,576) (Grarup et al. 2013), and folate (*n* = 64,979) were sourced from the UK Biobank Consortium or GWAS studies/meta‐analyses on individuals of European ancestry. Details of the exposure and outcome GWAS summary‐level data are provided in Table 1. All data were obtained from publicly available databases with ethical approval for each cohort, obviating the need for additional informed consent in this study.

**TABLE 1:** Details of the GWAS summary‐level data.

Phenotype	Consortium	Sample size	Ethnicity	Data accession address
Hcy (Exposure)	A meta‐analysis of GWAS	44,147	European	https://doi.org/10.3945/ajcn.112.044545
Vit B_6_ (Exposure)	UKBiobank	64,979	European	https://gwas.mrcieu.ac.uk/
Vit B_12_ (Exposure)	Sequencing studies using WGS	45,576	European	https://doi.org/10.1371/journal.pgen.1003530
Folate (Exposure)	UKBiobank	64,979	European	https://gwas.mrcieu.ac.uk/
Low grip strength	A meta‐analysis of GWAS	256,523	European	https://doi.org/10.1038/s41467‐021‐20918‐w
Walking pace	UKBiobank	459,915	European	https://gwas.mrcieu.ac.uk/
ALM	GWAS	450,243	European	https://doi.org/10.1038/s42003‐020‐01334‐0

Abbreviations: ALM, appendicular lean mass; Hcy, homocysteine.

For the cohort study component, the diagnosis of incident sarcopenia during the follow‐up period was based on the Asian Working Group for Sarcopenia (AWGS) 2019 criteria. All baseline data were obtained by uniformly trained personnel following standardized protocols to ensure methodological consistency. During the initial health examination, comprehensive baseline information was systematically documented, including demographic characteristics, current and past medical history, medication use, and family history. Anthropometric measurements were performed using standardized procedures established by the Health Management Department. Height, weight, and BMI were measured with a SK‐CK ultrasonic body composition analyzer, with participants standing upright without shoes and wearing light clothing until stable readings were obtained. Blood pressure was measured by designated staff using an OMRON HBP‐9021 sphygmomanometer after participants had rested quietly for at least 5 min; two measurements were taken and averaged. For laboratory analyses, fasting venous blood samples were collected in the morning after at least 8 h of fasting. Biochemical parameters, including uric acid (UA), fasting blood glucose (FBG), lipid profiles, and plasma Hcy levels (a key exposure variable in this study), were analyzed using an AU5800 series automated biochemical analyzer (Beckman Coulter, USA). For the assessment of sarcopenia‐related parameters, handgrip strength was measured by trained researchers using a dynamometer with participants in a standing position and elbows extended; tests were performed with the dominant hand or both hands, repeated at least twice, and the maximum value was recorded. Walking speed was assessed via a 6‐min walk test, where participants walked at their fastest pace on a flat, unobstructed surface, with the total distance converted to meters per second. ALM was quantified using dual‐energy X‐ray absorptiometry (DXA). The study protocol received ethical approval from the Institutional Review Board of the First Hospital of Shanxi Medical University (Approval No. KYLL‐2024‐081).

### 2.3 Selection of Instrumental Variables for MR Analysis

IVs for exposure and outcome should meet the following criteria: (1) Significant SNP associated with exposure are identified using a significance threshold of *p* < 5 × 10^−8^; (2) To satisfy the assumptions, LD‐related SNPs are removed by setting a linkage disequilibrium coefficient of *R* ^2^ = 0.001 within a region width of kb = 10,000; (3) Homopolymers and incompatible SNPs that could influence the causal relationship between allele variants and sarcopenia‐related traits are excluded; (4) The strength of IVs is assessed using the *F*‐statistic calculated through a formula:

$$F\;\text{statistic}=\left(\frac{\left(N-K-1\right)}{K}\right)\left(\frac{R^{2}}{1-R^{2}}\right),$$

with an *F*‐statistic > 10 considered to exclude weakly correlated SNP.

### 2.4 Grouping of Hcy Trajectories

The health examination data of four consecutive years from January 2018 to December 2021 were analyzed. Based on the group‐based trajectory model (GBTM), the number of groups of Hcy trajectory was determined according to the minimization principle of Bayesian information criterion (BIC) under the premise that the proportion of each group was more than 5%. According to the average posterior grouping probability (AvePP) > 0.7, the best trajectory model was determined and the degree of fit of the trajectory was evaluated (Nagin et al. 2024). Three different Hcy trajectory groups were generated, named as: Low stable group, medium stable group, and high stable group. The baseline characteristics and the risk of sarcopenia in each group were analyzed.

### 2.5 Statistical Analysis

MR analysis was conducted using the TwoSampleMR package in the R 4.4.1 version software. The MR‐Egger, Weighted median, inverse variance weighted (IVW), Simple mode, Weighted mode and MR‐Egger (bootstrap) methods were used as the default analysis methods. Among the methods employed, the IVW method was designated as our primary analytical approach, as it provides the most precise estimate under the assumption that all genetic variants are valid instruments. The MR‐Egger, weighted median, simple mode, weighted mode and MR‐Egger (bootstrap) methods were used as complementary analyses. The robustness of the primary IVW result was rigorously evaluated by examining the consistency in both the direction and magnitude of the causal estimates across all Supporting Information. A statistically significant IVW result that was consistently supported by the estimates from the other methods was interpreted as strong evidence for a causal relationship. Conversely, notable discrepancies particularly instances where a significant other methods result was not corroborated by the IVW methods‐suggested that the primary finding might be biased by horizontal pleiotropy and were therefore interpreted with considerable caution. Causal associations between exposure and outcome were assessed based on *p*‐values (*p* < 0.05 indicating statistical significance). Cochran's *Q* test evaluated heterogeneity among IVs (*p* < 0.05 indicating significant heterogeneity), while MR Egger regression was used to test for the presence of level pleiotropy. Intercept in Egger regression is an effective indicator of whether the results of MR Analysis are affected by level pleiotropy. If intercept is not equal to zero, it indicates that there is overall level pleiotropy. The leave‐one‐out method was employed to conduct sensitivity analysis, whereby each SNP was sequentially eliminated and the effect size of the remaining SNPs was calculated. If the exclusion of any single SNP significantly impacts the results, it indicates the presence of sensitivity.

Stata18.0 and SPSS26.0 statistical software were used to analyze the data. Measurement data were expressed as mean ± SD, and one‐way analysis of variance was used for comparison among groups. Count data were expressed as frequency (%), and chi‐square test was used for comparison between groups. The Hcy trajectory model of the subjects was established by Traj program and grouped to determine the best number of trajectory groups and select the best trajectory model (Nagin and Odgers 2010). The Kaplan–Meier method was used to calculate the cumulative incidence of sarcopenia in different groups, and the Log‐rank test was used to compare the difference in the cumulative incidence of sarcopenia in each group. Cox proportional hazards regression model was used to analyze the association between different Hcy trajectories and the risk of sarcopenia. A total of three models were established in this study. Model 1 did not adjust any variables. Model 2 was adjusted for age, sex, and BMI. Model 3 was further adjusted on the basis of Model 2 for exercise, smoking, drinking, systolic blood pressure (SBP), diastolic blood pressure (DBP), UA, FBG, TC, TG, high‐density lipoprotein cholesterol (HDL‐C), and low‐density lipoprotein cholesterol (LDL‐C). The restricted cubic spline regression model was established to evaluate the dose–response relationship between Hcy and sarcopenia. The number of nodes was selected as four to fit the restricted cubic spline function, and the dose–response relationship was plotted after adjusting for age, gender, BMI, exercise, drinking, SBP, UA, FBG.

## 3 Results

### 3.1 MR Analyses

#### 3.1.1 Selection of Instrumental Variables

We screened a total of 63 independent SNP instruments and ultimately identified 51 usable SNPs. In the MR analysis, the effects of SNP on exposure and the effects of SNP on outcomes must correspond to the same allele. After coordinating the exposure and outcome data, deleting duplicates and inversely correlated SNPs, Hcy, Vit B_6_, folate (Vit B_9_), and Vit B_12_ had 10, 17, 13, and 11 instruments, respectively (Tables S2 and S3).

#### 3.1.2 Results of Causal Effects Between Hcy Levels, B Vitamins on Sarcopenia‐Related Traits

**FIGURE 2:**
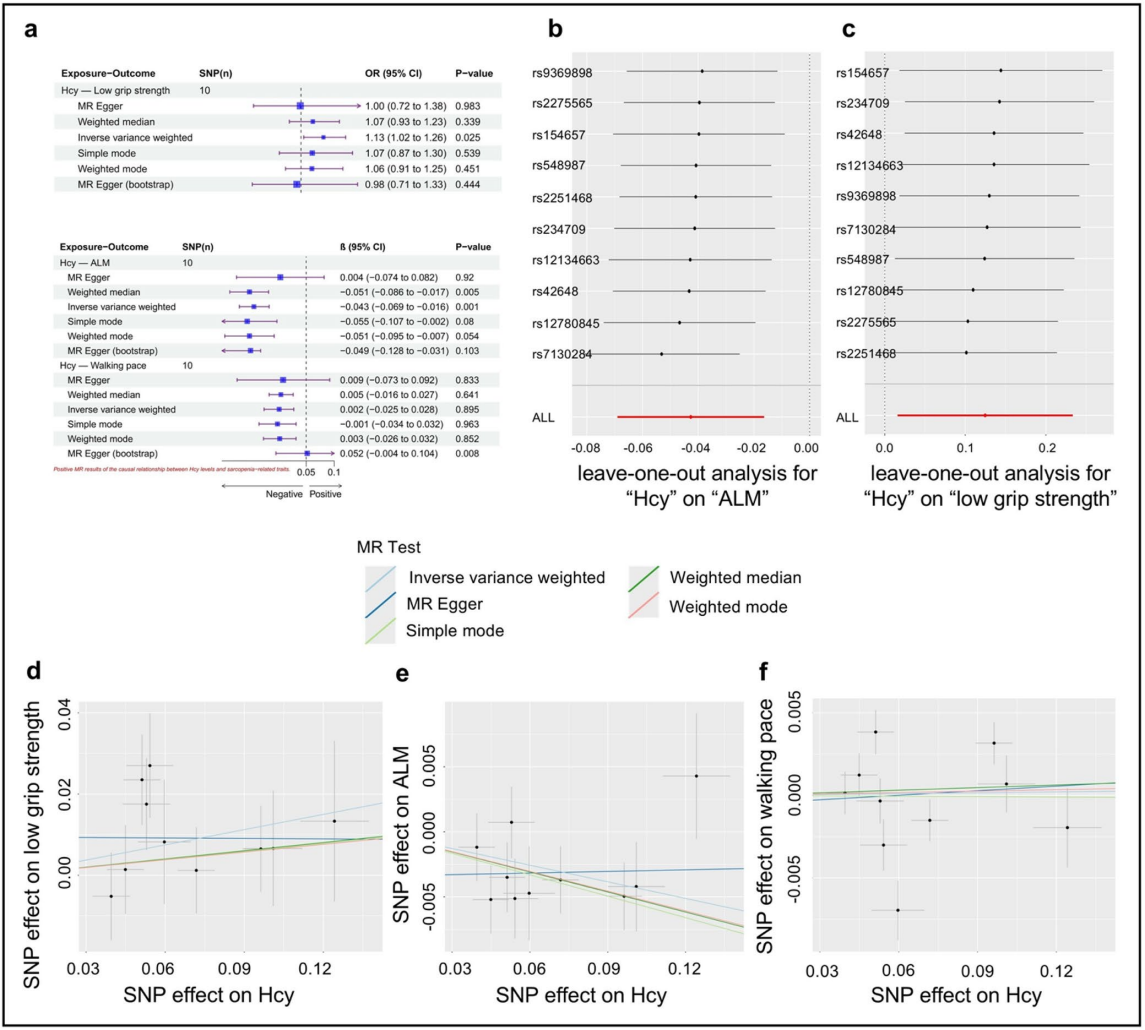
(a) Causal effect of Hcy on sarcopenia‐related traits in MR analyses. (b) Leave‐one‐out sensitivity analysis for the Hcy‐ALM association. (c) Leave‐one‐out sensitivity analysis for the Hcy‐low grip strength association. (d) Scatter plot showing the association between Hcy and low grip strength. (e) Scatter plot showing the association between Hcy and ALM. (f) Scatter plot showing the association between Hcy and walking pace.

Figure 2a displays the results of MR analyses exploring the association between serum Hcy levels and sarcopenia‐related traits. The MR analysis conducted primarily using the IVW method showed a significant causal relationship between genetically predicted Hcy levels and low grip strength (OR = 1.133, 95% CI = 1.016–1.263, *p* = 0.025). This result was directionally consistent with estimates from sensitivity analyses, including the weighted median (OR = 1.07, 95% CI: 0.93–1.23), Simple mode (OR = 1.07, 95% CI: 0.87–1.30), Weighted mode (OR = 1.06, 95% CI: 0.91–1.25) and MR‐Egger methods, though these did not reach statistical significance. Similarly, genetically predicted Hcy levels were also found to have a significant causal relationship with ALM (*β* = −0.043, 95% CI = −0.069 to −0.016, *p* = 0.001), indicating that each one‐standard deviation increase in Hcy levels was associated with a 0.043 standard deviation decrease in ALM. This finding was corroborated by the weighted median and simple mode estimators, which yielded effects in the same direction. Based on the results of the IVW method, we did not find a significant causal association between genetically predicted Hcy levels and walking pace. Although the MR Egger (bootstrap) analysis showed a borderline significant positive association (*β* = 0.052, 95% CI = −0.004 to 0.104, *p* = 0.008), this finding is not conclusive, given that its confidence interval contains zero values and is inconsistent with the results of other analytical methods.

In the Weighted median method, there was evidence of a significant causal relationship between Vit B_6_ and Walking pace (*β* = 0.037, 95% CI = 0.001–0.073, *p* = 0.038, Figure S4). However, further sensitivity analysis and results from a random‐effects model led to an insignificant association (*p* > 0.05, Table S5). Additionally, MR analysis showed no significant association between Vit B_6_ and low grip strength or ALM (all *p* > 0.05). The results of the MR Egger (bootstrap) method used as a reference reveal a causal association between Vit B_12_ and sarcopenia‐related traits, with estimated (*β* = −0.038, 95% CI = −0.064 to 0.003, *p* = 0.002) for ALM, (OR = 1.107, 95% CI = 0.979 to 1.252, *p* = 0.048) for low grip strength, and (*β* = 0.024, 95% CI = 0.006 to 0.042, *p* = 0.002) for walking pace (Figure S5). However, the IVW and other default methods did not find this association. For serum folate (Vit B_9_) levels, although we used the default method and supplemented it with the MR Egger (bootstrap) method, all methods yielded negative results between Vit B_9_ and sarcopenia‐related traits. The corresponding results are listed in Table S3. In conclusion, the evidence regarding the causal role of B vitamins in sarcopenia remains unclear, and due to methodological inconsistencies and the lack of strong support in both the primary analysis and sensitivity analyses, the results must be interpreted with caution.

#### 3.1.3 Sensitivity Analysis

For the association between serum Hcy levels and low grip strength and ALM, the results of sensitivity analysis and level pleiotropy detection were all *p* > 0.05, thus confirming the stability of our findings (Tables S4 and S6). In the graphical results, we plotted a forest plot to demonstrate the influence of individual SNP on causal estimates (Figure 2d–f). The final scatter plot results showed a uniform distribution. Furthermore, the leave‐one‐out plot analysis of the MR analysis between serum Hcy levels and low grip strength and ALM also showed that the results were stable and not affected by any single SNP (Figure 2b,c). Cochrane's *Q* test indicated that heterogeneity was observed in the analysis of Vit B_6_ versus walking pace. We then reanalyzed the analysis in a random‐effects model, which yielded a negative result (*p* = 0.944). Regarding the positive MR results obtained by the MR Egger (bootstrap) reference method, further sensitivity analyses were not performed due to method limitations.

### 3.2 Longitudinal Trajectory Analysis

#### 3.2.1 The Situation of the Hcy Trajectory Group

**FIGURE 3:**
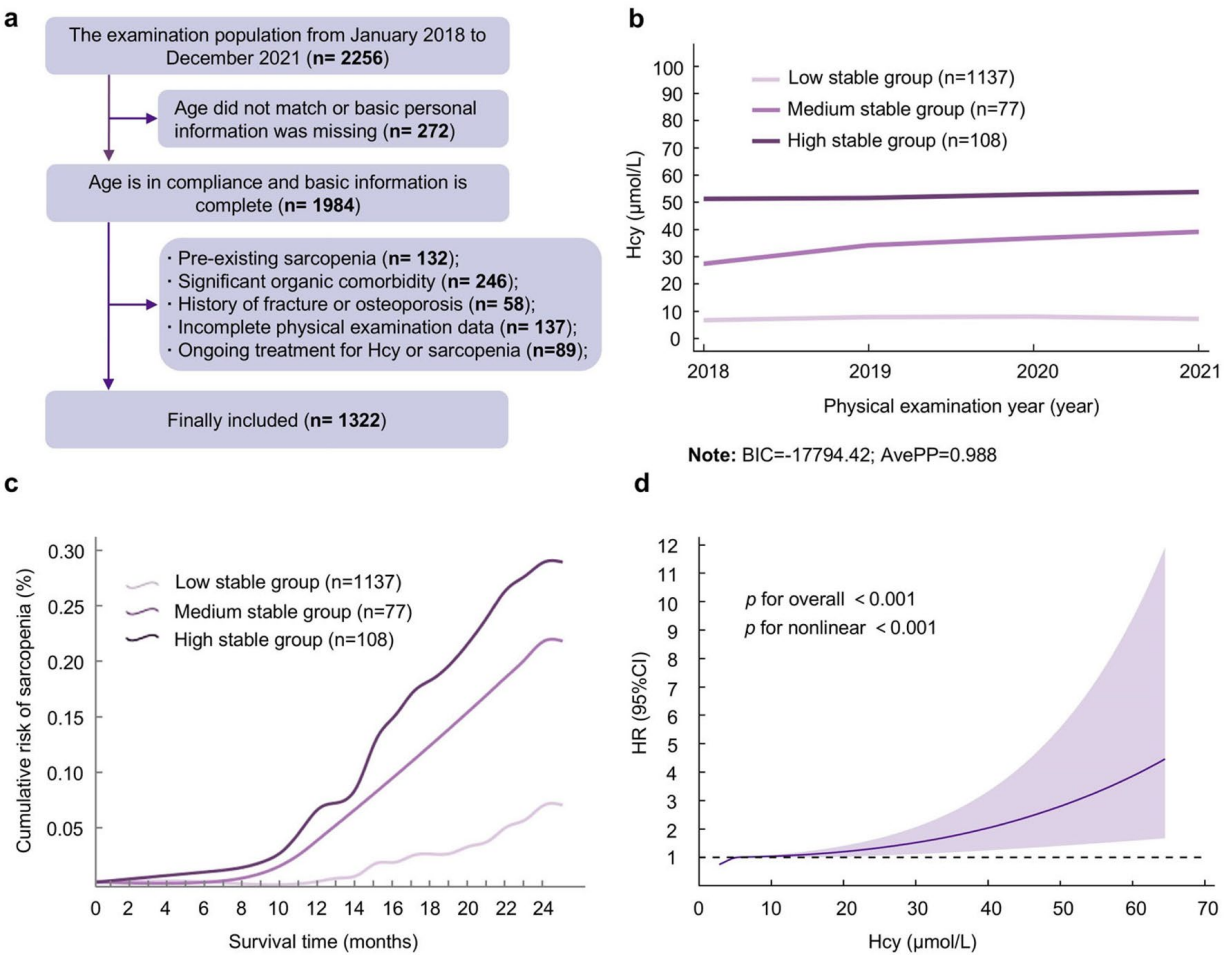
Participant inclusion flowchart, Hcy trajectory groups, and their association with sarcopenia risk. (a) Flowchart of study participant selection. (b) Identified Hcy trajectory groups. (c) Kaplan–Meier curves for cumulative risk of sarcopenia across trajectory groups. (d) Dose–response relationship between serum Hcy levels and sarcopenia risk. This curve is plotted based on a 4‐node restricted cubic spline regression model. The model takes into account variables such as age, gender, exercise, smoking, drinking, BMI, SBP, DBP, UA, FBG, TC, TG, HDL‐C, and LDL‐C as confounding variables. The black horizontal dotted line represents the reference line when the HR value is 1, the purple solid line represents the HR value, and the shaded area represents the 95% CI.

This study conducted a long‐term follow‐up on 1322 participants (average age 36.5 ± 8.4 years, 51.1% male). Figure 3a illustrates the screening and inclusion process of the participants. Based on the trajectory of serum Hcy levels, three subgroups were determined: the low‐stable group (*n* = 1137, 86.0%), the medium‐stable group (*n* = 77, 5.8%), and the high‐stable group (*n* = 108, 8.2%) (Figure 3b). During the follow‐up period from 2022 to 2023, a total of 118 new cases of sarcopenia were identified. Survival analysis showed that the cumulative incidence rates in the low, medium, and high stable groups showed a significant increasing trend, respectively, 6.68%, 19.48%, and 25.00% (Log‐rank *χ* ^2^ = 59.638, *p* < 0.001; Figure 3c).

Baseline characteristic analysis revealed significant differences among the three groups in terms of age, gender, exercise, smoking, drinking, BMI, UA, FBG, blood lipids, etc. (*p* < 0.05). The incidence of sarcopenia increased with the elevation of Hcy levels (Table S7). Notably, when comparing the participants with and without sarcopenia within each trajectory group, the baseline age of those who eventually developed sarcopenia was significantly higher than that of the non‐affected individuals in all three trajectory groups (Table 2). Additionally, gender and exercise patterns also showed significant associations. In the low‐stable and medium‐stable groups, the proportion of females in the sarcopenia subgroup was higher; while in the high‐stable group, the proportion of those who were “inactive” or “occasionally active” at baseline was significantly higher in the sarcopenia subgroup (81.5%). In terms of metabolic indicators, the baseline BMI of participants with sarcopenia was significantly lower in the low and high stable groups, while SBP, DBP, and UA levels were generally higher.

**TABLE 2:** Comparison of baseline data between non‐sarcopenia group and sarcopenia group in different Hcy trajectory groups.

Variables	Low‐stable group		Medium‐stable group		High‐stable group	
	Non‐Sar group (*n* = 1061)	Sar group (*n* = 76)	Non‐Sar group (*n* = 62)	Sar group (*n* = 15)	Non‐Sar group (*n* = 81)	Sar group (*n* = 27)
Age (years, Mean ± SD)	35.4 ± 6.9	44.7 ± 11.8^a^	38.2 ± 8.7	51.7 ± 16.8^a^	32.5 ± 4.8	54.3 ± 9.9^a^
Sex [*n* (%)]						
Male	604 (56.9)	26 (34.2)	10 (16.1)	13 (86.7)	13 (16.0)	10 (37.0)
Female	457 (43.1)	50 (65.8)^a^	52 (83.9)	2 (13.3)	68 (84.0)	17 (63.0)
Physical Activity [*n* (%)]						
None/Sedentary	202 (19.0)	23 (30.3)	28 (45.2)	2 (13.3)	15 (18.5)	16 (59.3)
Occasional	575 (54.2)	47 (61.8)	26 (41.9)	7 (46.7)	39 (48.1)	6 (22.2)^a^
Regular	284 (26.8)	6 (7.9)^a^	8 (12.9)	6 (40.0)	27 (33.3)	5 (18.5)^a^
Smoking Status [*n* (%)]						
Never	507 (47.8)	30 (39.5)	19 (30.6)	9 (60.0)	35 (43.2)	3 (11.1)
Occasional	362 (34.1)	29 (38.2)	18 (29.0)	5 (33.3)	19 (23.5)	14 (51.9)^a^
Current	187 (17.6)	11 (14.5)	11 (17.7)	1 (6.7)	19 (23.5)	9 (33.3)
Former	5 (0.5)	6 (7.9)^a^	14 (22.6)	0 (0.0)	8 (9.9)	1 (3.7)
Alcohol consumption [*n* (%)]						
Never	245 (23.1)	11 (14.5)	31 (50.0)	10 (66.7)	40 (49.4)	2 (7.4)
Occasional	261 (24.6)	21 (27.6)^a^	7 (11.3)	5 (33.3)	7 (8.6)	11 (40.7)^a^
Current	450 (42.4)	43 (56.6)^a^	20 (32.3)	0 (0.0)	24 (29.6)	14 (51.9)^a^
Former	105 (9.9)	1 (1.3)	4 (6.5)	0 (0.0)	10 (12.3)	0 (0.0)
BMI, (kg/m^2^, mean ± SD)	24.41 ± 3.17	23.55 ± 3.11^a^	23.72 ± 3.16	23.01 ± 3.02	24.11 ± 3.01	20.79 ± 1.43^a^
SBP (mmHg, mean ± SD)	118.68 ± 14.26	136.63 ± 19.20^a^	117.33 ± 14.04	113.40 ± 12.57	116.05 ± 11.88	138.44 ± 23.48^a^
DBP (mmHg, mean ± SD)	74.49 ± 8.95	85.83 ± 14.08^a^	74.77 ± 9.08	62.93 ± 4.35^a^	71.80 ± 9.49	83.26 ± 13.02^a^
UA (μmol/L, mean ± SD)	333.01 ± 87.38	385.18 ± 114.17^a^	317.44 ± 100.12	312.80 ± 106.07	269.95 ± 59.51	323.93 ± 117.12^a^
FBG (mmol/L, mean ± SD)	5.35 ± 1.25	6.74 ± 2.59^a^	4.95 ± 0.53	7.80 ± 2.98^a^	5.01 ± 0.65	5.03 ± 0.60
TC (mmol/L, mean ± SD)	5.01 ± 1.03	4.98 ± 0.99	4.91 ± 0.93	4.87 ± 1.39	4.71 ± 1.00	5.08 ± 1.09
TG (mmol/L, mean ± SD)	1.75 ± 1.11	1.71 ± 1.23	1.29 ± 0.67	1.83 ± 1.22^a^	1.51 ± 1.00	1.10 ± 0.51^a^
LDL‐C (mmol/L, mean ± SD)	3.27 ± 0.91	3.09 ± 0.73	3.03 ± 0.62	3.18 ± 0.94	2.97 ± 0.71	3.15 ± 0.73
HDL‐C (mmol/L, mean ± SD)	1.32 ± 0.27	1.45 ± 0.37^a^	1.26 ± 0.33	1.29 ± 0.33	1.05 ± 0.28	0.92 ± 0.22^a^

Abbreviations: Non‐sar group, non‐sarcopenia group; Sar group, sarcopenia group.

^a^ Compared with non‐sarcopenia group, *p* < 0.05.

#### 3.2.2 Cox Regression Model for the Risk of Sarcopenia Onset After Stepwise Adjustment

After adjusting for age, gender, BMI, exercise, smoking, drinking, SBP, DBP, UA, FBG, TG, triglycerides, LDL‐C, and HDL‐C, compared with the low‐stability group, the risk of sarcopenia in the medium‐stable group and high‐stable group increased by 1.965 times and 2.832 times, respectively (all *p* < 0.05) (Table 3).

**TABLE 3:** Cox regression risk model for the occurrence of sarcopenia in different Hcy trajectory groups.

Group	Model 1		Model 2		Model 3	
	HR (95% CI)	*p*	HR (95% CI)	*p*	HR (95% CI)	*p*
Low‐stable group	1		1		1	
Medium‐stable group	3.202 (1.840–5.571)	< 0.001	1.110 (0.601–2.052)	0.739	1.965 (1.027–3.759)	0.041
High‐stable group	4.315 (2.781–6.695)	< 0.001	2.711 (1.673–4.393)	< 0.001	2.832 (1.608–4.987)	< 0.001

*Note:* The low stability group was used as the reference group. Model 1 took different Hcy trajectory groups as the independent variable (the low‐stable group as the reference group) and the presence or absence of sarcopenia as the dependent variable (defined as 0 without sarcopenia and 1 with sarcopenia). Model 2 adjusted age, gender, and BMI on the basis of Model 1. Model 3 was adjusted on the basis of Model 2 for exercise, smoking, drinking, SBP, DBP, UA, FBG, TC, TG, LDL‐C, and HDL‐C. Multicollinearity tests have been performed (all VIFs < 3).

#### 3.2.3 Dose–Response Relationship Between Baseline Hcy and the Risk of Sarcopenia

Furthermore, after adjusting for confounding factors, there was a nonlinear dose–response relationship between Hcy and the risk of sarcopenia onset (*p* nonlinear < 0.001). When Hcy levels were > 12.5 μmol/L, the risk of sarcopenia gradually increased with the elevation of Hcy levels (Figure 3d).

## 4 Discussion

This study employed an integrated analytical approach combining MR with longitudinal cohort data to systematically evaluate the relationships between Hcy, B vitamins, and sarcopenia‐related traits. It is important to clarify that the MR analysis aimed to investigate the potential causal effects on core sarcopenia‐related phenotypes (grip strength, walking pace, and ALM), whereas the longitudinal cohort was used to validate the association with clinically diagnosed sarcopenia. The consistency of causal directions across multiple MR methods with different underlying assumptions—particularly the close concordance between IVW, weighted median, and mode‐based estimates for the effects of Hcy on grip strength and ALM—significantly strengthens the credibility of these genetic findings. MR analyses demonstrated a significant causal association between elevated Hcy levels and sarcopenia characteristics, supported by robust sensitivity analyses controlling for heterogeneity and horizontal pleiotropy. These genetic findings were corroborated by longitudinal observations, where Kaplan–Meier survival analysis revealed significantly different cumulative incidence of sarcopenia across Hcy trajectory groups (log‐rank *p* < 0.001). Furthermore, multivariable Cox proportional hazards models confirmed substantially increased sarcopenia risk in medium and high Hcy trajectory groups after comprehensive adjustment for confounders (HR = 2.965, 95% CI: 1.687–5.212; HR = 3.832, 95% CI: 2.405–6.105). Subgroup analyses consistently identified advanced age, female sex, physical inactivity, and metabolic disorders as significant risk factors across all Hcy trajectory groups. Regarding B vitamins, the evidence remained inconclusive. While the weighted median method suggested a potential association between Vit B_6_ and sarcopenia traits, this finding was not supported by sensitivity analyses using random‐effects models. Similarly, although some analytical approaches indicated possible relationships involving Vit B_12_, these results were characterized by methodological limitations and inconsistent findings across sensitivity analyses, precluding any firm causal conclusions. No robust genetic evidence supported causal roles for folate in sarcopenia.

### 4.1 Comparison With Previous Studies

To the best of our knowledge, this is the first study to systematically evaluate the association between Hcy, B vitamins and sarcopenia using a MR design with long‐term longitudinal data. The association between serum Hcy levels and sarcopenia or its traits has been studied in many studies, but there have been conflicting results. A cross‐sectional study of 114,583 community‐dwelling adults in Korea found that high Hcy levels were significantly associated with low skeletal muscle mass (LMM), with a 1.360‐fold and 2.169‐fold increase in the risk of mild and severe LMM, respectively, for each unit increase in Hcy levels, which is consistent with the results of this study (Choi et al. 2022). Another study of 441 elderly Chinese participants also found that serum Hcy levels was significantly associated with sarcopenia after adjusting for age, sex, etc. (with ASMI < 7.0 kg/m^2^ for men and ASMI < 5.7 kg/m^2^ for women) (Lu et al. 2022). In summary, an elevated Hcy levels is significantly associated with decreased skeletal muscle mass and function. However, there are also studies that show no significant association between the two, which is our biggest concern. A study from the UK that pooled 85 or more studies, including 845 elderly participants, found that neither a cross‐sectional study nor a 5‐year prospective follow‐up showed an association between Hcy levels and grip strength (Granic et al. 2017). Furthermore, a clinical study from Japan indicated no significant association between Hcy levels and sarcopenia (Eguchi et al. 2021). We attribute the discrepancy between previous and current studies to differences in study type, design, and ethnicity. The MR Design used in this study was able to effectively control for confounding factors and reverse causality, and the long‐term longitudinal data further provide evidence of the temporal association, which together strengthen the potential causal role of Hcy in muscle health.

### 4.2 Possible Explanations

It is worth noting that before these associations can be translated into clinical practice evidence, further research into the underlying mechanisms is crucial. Hcy is a sulfur‐containing amino acid, but not one of the 20 essential amino acids, which is synthesized from methionine and then converted through the remethylation pathway and the transsulfuration pathway (Blom and Smulders 2011). Under normal conditions, the serum Hcy level is 5–15 μmol/L. Due to problems with protease, lack of cofactors, excessive intake of methionine, folate and B vitamins deficiency, and taking certain drugs, Hcy levels can be elevated (Wierzbicki 2007; Wang et al. 2022). The most important thing is that there are multiple potential mechanisms by which elevated Hcy levels are associated with reduced muscle mass and function, including oxidative stress, protein aggregation, functional disorder, stimulation of cell apoptosis, mitochondrial dysfunction, and promotion of inflammatory responses, leading to dysregulation of the muscle system (Vidoni et al. 2018).

First, serum Hcy primarily damages skeletal muscle through the oxidative stress process (Veeranki, Winchester, and Tyagi 2015; Veeranki, Lominadze, and Tyagi 2015; Majumder et al. 2017). When the body is in high Hcy levels, the levels of endoplasmic reticulum stress markers are significantly increased, the level of malondialdehyde, a lipid peroxidation end product, is increased, the activity of antioxidant enzymes such as superoxide dismutase and glutathione peroxidase is reduced, and oxidative stress and antioxidant imbalance lead to muscle atrophy (Majumder et al. 2018; Yakovleva et al. 2018). In addition, mitochondrial dysfunction will also reduce the stability and tolerance of skeletal muscle, which is closely related to muscle atrophy (Chen et al. 2010). Veeranki, Lominadze, and Tyagi (2015) and Veeranki, Winchester, and Tyagi (2015) found that muscle weakness and fatigue are not due to changes in key enzymes involved in metabolism, but mainly due to a decrease in ATP levels. Finally, Hcy can affect skeletal muscle changes by inducing the expression of various inflammation‐related factors, such as the expression of matrix metalloproteinase‐9 (MMP‐9) increasing, while MMP‐9 is an intracellular proteolytic enzyme that degrades collagen in the extracellular matrix, which can trigger tissue remodeling and lead to muscle fibrosis (Winchester et al. 2018). Animal studies also found that during high Hcy blood levels, the expression of connective tissue protein in skeletal muscle vasculature would decrease, leading to an increase in myostatin expression (Givvimani et al. 2013; Looft‐Wilson et al. 2004). And myostatin not only inhibits the growth of muscle cells but also directly regulates muscle fibroblasts, leading to muscle fibrosis and atrophy (Li et al. 2008).

Currently, there is growing interest in the relationship between nutrients and muscle function and quality (El‐Sayed et al. 2024; Akehurst et al. 2024). Micronutrients include amino acids and their derivatives, vitamins, probiotics, and minerals (Shen et al. 2024). Among them, B vitamins play indispensable roles in maintaining muscle health by regulating mitochondrial biogenesis, redox homeostasis, protein metabolism, and neuromuscular function (Aytekin et al. 2018; Kato et al. 2024). In this study, we aimed to determine whether B vitamins are significantly associated with sarcopenia and its associated features, but the results were mixed. Our findings did not reach statistical significance, possibly due to the relatively limited number of genetic tools utilized in the study. We cannot entirely exclude the possibility that our study may lack sufficient power to detect subtle associations. Nonetheless, we also contribute to the exploration of the association of micronutrients related to sarcopenia‐related traits.

### 4.3 Strengths and Limitations

This study possesses several notable strengths. First, the integration of a two‐sample MR design with an independent longitudinal cohort provides a robust and complementary analytical framework. The MR component leverages genetic variants to infer causality while minimizing confounding, whereas the longitudinal analysis offers critical clinical validation by establishing temporal and dose–response relationships. Second, the use of multiple, complementary MR methods (IVW, weighted median, MR‐Egger, etc.) and rigorous sensitivity analyses (e.g., MR‐PRESSO test, “leave‐one‐out” analysis) enhances the reliability of our causal inferences and allows for a thorough assessment of potential pleiotropy. Third, our longitudinal cohort analysis employed advanced GBTM to capture the dynamic progression of Hcy over 4 years, providing a more nuanced view of exposure than a single baseline measurement. Finally, the comprehensive adjustment for a wide array of confounders in the Cox models and the exploration of nonlinear dose–response relationships strengthen the clinical relevance and validity of our observational findings.

However, several limitations must be acknowledged. First, it should be recognized that heterogeneity cannot be completely eliminated due to the inherent limitations of MR methods. Second, the genetic discovery and instrumental variables in our MR analysis were based on individuals of European ancestry, whereas the clinical validation cohort comprised an Asian population. Differences in genetic background, lifestyle, and environmental factors across ancestries may influence the generalizability of our findings. Therefore, the conclusions need to be further validated in non‐European populations and across diverse ethnic groups. Third, the lack of individual‐level information in the pooled data hindered our ability to further investigate the influence of factors such as gender, lifestyle, or occupation on the established associations. Furthermore, although the longitudinal cohort analysis provides long‐term follow‐up evidence with clinical relevance and adjusts for multiple confounding factors including demographic characteristics and metabolic factors in the model, there may still be other confounding factors. The conclusions drawn from this study also require external validation in larger GWAS samples or in a clinical setting.

## 5 Conclusion and Future Perspectives

In conclusion, this integrated MR and longitudinal study provides robust evidence supporting a causal and temporal relationship between elevated Hcy levels and an increased risk of sarcopenia‐related traits. The genetic evidence from MR analyses was corroborated by clinical data showing a clear dose–response relationship and significantly higher sarcopenia incidence in individuals with sustained medium or high Hcy trajectories. Conversely, our findings did not yield consistent or robust genetic evidence to support causal roles for folate, vit B_6_, or vitamin B_12_ in sarcopenia etiology within the studied populations. These findings highlight Hcy as a promising biomarker and potential modifiable risk factor for muscle health. Future research should focus on elucidating the underlying mechanisms and evaluating the efficacy of Hcy‐lowering strategies in preventing sarcopenia.

Our findings highlight plasma Hcy as a promising biomarker for muscle health assessment. From a clinical perspective, integrating Hcy monitoring into geriatric evaluations could help identify older adults at elevated risk for sarcopenia, enabling timely interventions. Regarding nutritional implications, while our study provides a biological rationale for maintaining adequate B vitamins, it is crucial to emphasize that our MR analysis did not yield consistent evidence to directly support B vitamins supplementation for sarcopenia prevention. The key translational hypothesis arising from this work is that Hcy‐lowering strategies—which may include dietary optimization or targeted supplementation—represent a viable target for future interventional research. To advance this field, future studies should prioritize large‐scale, multi‐ethnic cohorts to validate and generalize these causal associations and employ multi‐omics approaches to elucidate the underlying mechanisms. Ultimately, this hypothesis requires rigorous testing in long‐term, randomized controlled trials to determine whether such strategies can effectively preserve muscle mass and function in at‐risk populations.

## Author Contributions

**Caizheng Yang:** data curation (equal), formal analysis (equal), software (equal), writing – original draft (equal). **Shanshan Ge:** project administration (equal), resources (equal), supervision (equal), writing – review and editing (equal). **Fangying Tian:** project administration (equal), resources (equal), supervision (equal), writing – review and editing (equal). **Yan Jiang:** conceptualization (equal), investigation (equal), methodology (equal), resources (equal), writing – original draft (equal). **Yue Guo:** writing – original draft (equal). **Xiumei Wang:** project administration (equal), resources (equal), supervision (equal), writing – review and editing (equal). **Hongwei Wang:** data curation (equal), formal analysis (equal), software (equal), visualization (equal), writing – original draft (equal), writing – review and editing (equal).

## Funding

This study was supported by the Science and Technology Innovation Project of Colleges and Universities in Shanxi Province [Grant numbers: 2024L090]; the Research project of High‐quality Development in Shanxi Province [Grant numbers: SXGZL202423]; and the Research Project of Science and Technology Innovation Think Tank Construction of Shanxi Association for Science and Technology [Grant numbers: KXKT202318].

## Ethics Statement

Ethical approval for the observational study of this research was granted by the Ethics Committee of the First Hospital of Shanxi Medical University [Approval No. KYLL‐2024‐081]. A waiver of ethical approval was granted for the Mendelian randomization analysis since it relied exclusively on publicly available summary data.

## Consent

The authors have nothing to report.

## Conflicts of Interest

The authors declare no conflicts of interest.

## Supporting information


**Figure S1:** Scatter plot of statistically significant results with IVW or default method as the primary method. (A) Hcy and hand grip strength. (B) Hcy and ALM. (C) Vit B_6_ and Walking pace.
**Figure S2:** Funnel plot of statistically significant results with IVW or default method as the primary method. (A) Hcy and hand grip strength. (B) Hcy and ALM. (C) Vit B_6_ and Walking pace.
**Figure S3:** Leave‐one‐out plots of statistically significant results with IVW or default method as the primary method. (A) Hcy and hand grip strength. (B) Hcy and ALM. (C) Vit B_6_ and Walking pace.
Figure **S4**. Causal effect of VB_6_ on sarcopenia‐related traits in MR analyses.
**Figure S5:**. Causal effect of VB_12_ on sarcopenia‐related traits in MR analyses.
**Table S1:** STROBE‐MR checklist of the present study.
**Table S2:** Details of instrumental variables.
**Table S3:** Associations of genetic prediction of serum levels of folate, Vit B_6_, Vit B_12_ and homocysteine (Hcy) with sarcopenia‐related traits in the MR‐Egger analysis.
**Table S4:** Heterogeneity of MR analysis for serum levels of folate, Vit B_6_, Vit B_12_, Hcy, and sarcopenia‐related traits.
**Table S5:** Effect sizes for Vit B_6_ and Walking pace were estimated using a random‐effects model.
**Table S6:** Associations of genetic prediction of serum levels of folate, Vit B_6_, Vit B_12_, and Hcy with sarcopenia‐related traits in the MR‐Level pleiotropy analysis.
**Table S7:** Comparison Table of Baseline Data of Different Hcy Trajectory Groups of 1322 Physical Examination Participants.

## Data Availability

The R code used for data analysis in this study has been deposited in the Gitee repository: https://gitee.com/hongwei‐wang10/MR‐LS‐r‐project. The summary‐level data for the MR analysis were obtained from publicly available GWAS, as cited in the manuscript. The individual‐level data from the longitudinal cohort study contain sensitive patient information and are not publicly available to protect patient privacy.
