# Supplementary material for: Genetically Predicted Homocysteine Levels and B Vitamins on Sarcopenia‐Related Traits: Insights From an Observational and Mendelian Randomization Analysis
Source: Food Sci Nutr. 2026 Feb 15;14(2):e71556. doi: 10.1002/fsn3.71556 (PMC12907574; doi:10.1002/fsn3.71556)
Supplement: Supplementary file 1 — Figure S1: Scatter plot of statistically significant results with IVW or default method as the primary method. (A) Hcy and hand grip strength. (B) Hcy and ALM. (C) Vit B6 and Walking pace. Figure S2: Funnel plot of statistically significant results with IVW or default method as the primary method. (A) Hcy and hand grip strength. (B) Hcy and ALM. (C) Vit B6 and Walking pace. Figure S3: Leave‐one‐out plots of statistically significant results with IVW or default method as the primary method. (A) Hcy and hand grip strength. (B) Hcy and ALM. (C) Vit B6 and Walking pace. Figure S4. Causal effect of VB6 on sarcopenia‐related traits in MR analyses. Figure S5:. Causal effect of VB12 on sarcopenia‐related traits in MR analyses. Table S1: STROBE‐MR checklist of the present study. Table S2: Details of instrumental variables. Table S3: Associations of genetic prediction of serum levels of folate, Vit B6, Vit B12 and homocysteine (Hcy) with sarcopenia‐related traits in the MR‐Egger analysis. Table S4: Heterogeneity of MR analysis for serum levels of folate, Vit B6, Vit B12, Hcy, and sarcopenia‐related traits. Table S5: Effect sizes for Vit B6 and Walking pace were estimated using a random‐effects model. Table S6: Associations of genetic prediction of serum levels of folate, Vit B6, Vit B12, and Hcy with sarcopenia‐related traits in the MR‐Level pleiotropy analysis. Table S7: Comparison Table of Baseline Data of Different Hcy Trajectory Groups of 1322 Physical Examination Participants. [file FSN3-14-e71556-s001.docx]

**Genetically Predicted Homocysteine Levels and B Vitamins on Sarcopenia- Related Traits: Insights from an Observational and Mendelian Randomization Analysis**

**Supplementary Materials**

**Supplementary Table S1.** STROBE-MR checklist of the present study.

**Supplementary Table S2.** Details of instrumental variables.

**Supplementary Table S3.** Associations of genetic prediction of serum levels of folate, vitamin B6, vitamin B12 and Hcy with sarcopenia-related traits in the MR-Egger analysis.

**Supplementary Table S4.** Heterogeneity of MR analysis for serum levels of folate, vitamin B6, vitamin B12, Hcy and sarcopenia-related traits.

**Supplementary Table S5.** Effect sizes for Hcy, VB6 and Walking pace were estimated using a random effects model

**Supplementary Table S6.** Associations of genetic prediction of serum levels of folate, vitamin B6, vitamin B12 and Hcy with sarcopenia-related traits in the MR-Level pleiotropy analysis.

**Supplementary Table S7.** Comparison Table of Baseline Data of Different Homocysteine Trajectory Groups of 1322 Physical Examination Participants.

**Supplementary Figure S1.** Scatter plot of statistically significant results with IVW or default method as the primary method. (A) Hcy and hand grip strength. (B) Hcy and ALM. (C) VB6 and Walking pace.

**Supplementary Figure S2.** Funnel plot of statistically significant results with IVW or default method as the primary method. (A) Hcy and hand grip strength. (B)Hcy and ALM. (C) VB6 and Walking pace.

**Supplementary Figure S3.** Leave-one-out plots of statistically significant results with IVW or default method as the primary method. (A) Hcy and hand grip strength. (B) Hcy and ALM. (C) VB6 and Walking pace.

**Supplementary Figure S4.** Causal effect of VB6 on sarcopenia-related traits in MR analyses.

**Supplementary Figure S5.** Causal effect of VB12 on sarcopenia-related traits in MR analyses.

**Supplementary Table S1.** STROBE-MR checklist of the present study.

| **No.** | **Section** | **Checklist item** | **Relevant text from manuscript** |  |
| --- | --- | --- | --- | --- |
| 1 | Title and abstract | Indicate MR as the study’s design in the title and the abstract as a main purpose of the study | Title, Abstract |  |
| 2 | Background | Explain the scientific background and rationale for the reported study. Explain the exposure and a plausible potential causal relationship between exposure and outcome. Justify why MR is a helpful method to address the study question. | Introduction: Lines 51 to 70 |  |
| 3 | Objectives | State specific objectives clearly, including prespecified causal hypotheses. State that MR is a method that intends to estimate causal effects. | Introduction |  |
| 4a | Study design and data sources | Setting: Describe the study design (two-sample MR) and the underlying population . Describe the setting, locations, and relevant dates, including periods of recruitment, exposure, follow-up, and data collection. | Materials and methods: 2.1 Study design |  |
| 4b |  | Participants: Report the eligibility criteria and the sources and methods of selection of participants. Report the sample size and whether any power or sample size calculations were carried out prior to the main analysis. | Materials and methods: 2.2 Data Sources |  |
| 4c |  | Describe measurement, quality control, and selection of genetic variants. | Materials and methods: 2.3 Selection of Instrumental Variables |  |
| 4d |  | For each exposure, outcome, and other relevant variables, describe methods of assessment and diagnostic criteria for diseases. | Materials and methods: 2.2 Data Sources |  |
| 4e |  | Provide details of ethics committee approval and participant informed consent, if relevant. | Materials and methods: 2.2 Data Sources |  |
| 5 | Assumptions | Explicitly state the 3 core instrumental variable (IV) assumptions for the main analysis (relevance, independence, and exclusion restriction), as well assumptions for any additional or sensitivity analysis. | Materials and methods: 2.1 Study design |  |
| 6a | Statistical methods: main analysis | Describe how quantitative variables were handled in the analyses (i.e., scale, units, model) | Materials and methods: 2.4 MR Analysis and sensitivity analysis |  |
| 6b |  | Describe how genetic variants were handled in the analyses and, if applicable, how their weights were selected | Materials and methods: 2.4 MR Analysis and sensitivity analysis |  |
| 6c |  | Describe the MR estimator (e.g. two-stage least squares, Wald ratio) and related statistics. Detail the included covariates and, in case of two-sample MR, whether the same covariate set was used for adjustment in the two samples | Materials and methods: 2.4 MR Analysis and sensitivity analysis |  |
| 6d |  | Explain how missing data were addressed | N/A |  |
| 6e |  | If applicable, indicate how multiple testing was addressed | Materials and methods: 2.4 MR Analysis and sensitivity analysis |  |
| 7 | Assessment of assumptions | Describe any methods or prior knowledge used to assess the assumptions or justify their validity | Materials and methods: 2.4 MR Analysis and sensitivity analysis |  |
| 8 | Sensitivity analyses and additional analyses | Describe any sensitivity analyses or additional analyses performed (e.g. comparison of effect estimates from different approaches, independent replication, bias analytic techniques, validation of instruments, simulations) | Materials and methods: 2.4 MR Analysis and sensitivity analysis |  |
| 9a | Software and pre-registration | Name statistical software and package(s), including version and settings used | Name statistical software and package(s), including version and settings used |  |
| 9b |  | State whether the study protocol and details were pre-registered (as well as when and where) | N/A |  |
| 10a | Descriptive data | Report the numbers of individuals at each stage of included studies and reasons for exclusion. Consider use of a flow diagram | N/A |  |
| 10b |  | Report summary statistics for phenotypic exposure(s), outcome(s), and other relevant variables (e.g. means, SDs, proportions) | Supplementary Table S2. |  |
| 10c |  | If the data sources include meta-analyses of previous studies, provide the assessments of heterogeneity across these studies | N/A |  |
| 10d |  | For two-sample MR:  i. Provide justification of the similarity of the genetic variant-exposure associations between the exposure and outcome samples  ii. Provide information on the number of individuals who overlap between the exposure and outcome studies | N/A |  |
| 11a | Main results | Report the associations between genetic variant and exposure, and between genetic variant and outcome, preferably on an interpretable scale | Results: 3.2. Results of causal effects between Hcy levels, B vitamins on sarcopenia-related traits |  |
| 11b |  | Report MR estimates of the relationship between exposure and outcome, and the measures of uncertainty from the MR analysis, on an interpretable scale, such as odds ratio or relative risk per SD difference | Results: 3.2. Results of causal effects between Hcy levels, B vitamins on sarcopenia-related traits |  |
| 11c |  | If relevant, consider translating estimates of relative risk into absolute risk for a meaningful time period | N/A |  |
| 11d |  | Consider plots to visualize results (e.g. forest plot, scatterplot of associations between genetic variants and outcome versus between genetic variants and exposure) | Supplementary Figure S1-S3 |  |
| 12a | Assessment of assumptions | Report the assessment of the validity of the assumptions | N/A |  |
| 12b |  | Report any additional statistics (e.g., assessments of heterogeneity across genetic variants, such as *I^2^*, Q statistic or E-value) | Results: 3.2. Results of causal effects between Hcy levels, B vitamins on sarcopenia-related traits |  |
| 13a | Sensitivity analyses and additional analyses | Report any sensitivity analyses to assess the robustness of the main results to violations of the assumptions | Supplementary Table S4 |  |
| 13b |  | Report results from other sensitivity analyses or additional analyses | Supplementary Table S5 |  |
| 13c |  | Report any assessment of direction of causal relationship (e.g., bidirectional MR) | Supplementary Table S4 |  |
| 13d |  | When relevant, report and compare with estimates from non-MR analyses | Supplementary Table S4 |  |
| 13e |  | Consider additional plots to visualize results (e.g., leave-one-out analyses) | Supplementary Figure S3 |  |
| 14 | Key results | Summarize key results with reference to study objectives | Discussion: Lines 185 to 196 |  |
| 15 | Limitations | Discuss limitations of the study, taking into account the validity of the IV assumptions, other sources of potential bias, and imprecision. Discuss both direction and magnitude of any potential bias and any efforts to address them | Limitations of the Study |  |
| 16a | Interpretation | Meaning: Give a cautious overall interpretation of results in the context of their limitations and in comparison with other studies | Discussion: 4.1 Comparison with Previous Studies |  |
| 16b |  | Mechanism: Discuss underlying biological mechanisms that could drive a potential causal relationship between the investigated exposure and the outcome, and whether the gene-environment equivalence assumption is reasonable. Use causal language carefully, clarifying that IV estimates may provide causal effects only under certain assumptions | Discussion: 4.2 Possible Explanations |  |
| 16c |  | Clinical relevance: Discuss whether the results have clinical or public policy relevance, and to what extent they inform effect sizes of possible interventions | Discussion: 4.2 Possible Explanations |  |
| 17 | Generalizability | Discuss the generalizability of the study results (a) to other populations, (b) across other exposure periods/timings, and (c) across other levels of exposure | Discussion |  |
| 18 | Funding | Describe sources of funding and the role of funders in the present study and, if applicable, sources of funding for the databases and original study or studies on which the present study is based | Funding |  |
| 19 | Data and data sharing | Provide the data used to perform all analyses or report where and how the data can be accessed, and reference these sources in the article. Provide the statistical code needed to reproduce the results in the article, or report whether the code is publicly accessible and if so, where | Data availability statement |  |
| 20 | Conflicts of Interest | All authors should declare all potential conflicts of interest | Conflicts of Interest |  |

**Supplementary Table S2.** Details of instrumental variables.

| Exposure |  | SNP | CHR:POS | effect_allele | other_allele | eaf | beta | se | *p* |
| --- | --- | --- | --- | --- | --- | --- | --- | --- | --- |
| HCY | 1 | rs1801133 | 1:11856378 | A | G | 0.34 | 0.1583 | 0.007 | 4.34E-10 |
|  | 2 | rs2275565 | 1:237048676 | T | G | 0.21 | -0.0542 | 0.009 | 1.96E-10 |
|  | 3 | rs7422339 | 2: 211248752 | A | C | 0.33 | 0.0864 | 0.008 | 4.58E-27 |
|  | 4 | rs9369898 | 6:49382193 | A | G | 0.62 | 0.0449 | 0.007 | 2.17E-10 |
|  | 5 | rs7130284 | 11:89148372 | T | C | 0.07 | -0.1242 | 0.013 | 1.88E-20 |
|  | 6 | rs154657 | 16:89708096 | A | G | 0.47 | 0.0963 | 0.007 | 1.74E-43 |
|  | 7 | rs234709 | 21:44486964 | T | C | 0.45 | -0.0718 | 0.007 | 3.90E-24 |
|  | 8 | rs4660306 | 1:45978675 | T | A | 0.33 | 0.0435 | 0.007 | 2.33E-09 |
|  | 9 | rs548987 | 6:25869371 | C | G | 0.13 | 0.0597 | 0.01 | 1.12E-08 |
|  | 10 | rs42648 | 7:89977760 | A | G | 0.4 | -0.0395 | 0.007 | 1.97E-08 |
|  | 11 | rs1801222 | 10:17156151 | A | C | 0.34 | 0.0453 | 0.007 | 8.43E-10 |
|  | 12 | rs2251468 | 12:121405126 | A | C | 0.65 | -0.0512 | 0.007 | 1.28E-12 |
|  | 13 | rs838133 | 19:49259529 | A | C | 0.45 | 0.0422 | 0.007 | 7.48E-09 |
|  | 14 | rs12134663 | 1:11838646 | A | C | 0.8 | -0.101 | 0.011 | 2.54E-21 |
|  | 15 | rs12780845 | 10:17223244 | A | G | 0.65 | 0.0529 | 0.009 | 7.80E-10 |
|  | 16 | rs957140 | 11:89201627 | A | G | 0.45 | -0.045 | 0.008 | 2.43E-10 |
|  | 17 | rs12921383 | 16:89859753 | T | A | 0.87 | -0.09 | 0.014 | 8.22E-11 |
|  | 18 | rs2851391 | 21:44487404 | T | C | 0.47 | 0.056 | 0.008 | 1.70E-12 |
| VB6 | 1 | rs188211816 | 1:172810049 | A | G | 0.0295 | -0.0786 | 0.0163 | 1.40E-06 |
|  | 2 | rs155599 | 2:158307375 | C | T | 0.7052 | 0.0343 | 0.006 | 1.00E-08 |
|  | 3 | rs3772928 | 3:115406478 | C | T | 0.5754 | -0.0292 | 0.0055 | 1.30E-07 |
|  | 4 | rs141933624 | 4:91118956 | A | G | 0.0215 | -0.0897 | 0.0193 | 3.30E-06 |
|  | 5 | rs183178622 | 4:103988054 | T | C | 0.0183 | -0.0989 | 0.0207 | 1.70E-06 |
|  | 6 | rs77806858 | 5:153476869 | C | T | 0.0704 | -0.0505 | 0.0106 | 1.90E-06 |
|  | 7 | rs12198456 | 6:120371988 | T | C | 0.0187 | 0.0920 | 0.0197 | 3.10E-06 |
|  | 8 | rs74640671 | 8:21550838 | T | C | 0.0110 | -0.1272 | 0.0274 | 3.40E-06 |
|  | 9 | rs12412051 | 10:2601269 | C | G | 0.0344 | 0.0709 | 0.0150 | 2.20E-06 |
|  | 10 | rs12226112 | 11:14163360 | T | G | 0.3414 | 0.0283 | 0.0057 | 7.50E-07 |
|  | 11 | rs361294 | 11 103993635 | C | A | 0.6913 | -0.0274 | 0.0060 | 4.30E-06 |
|  | 12 | rs9560457 | 13 90476979 | T | C | 0.4042 | 0.0255 | 0.0055 | 4.10E-06 |
|  | 13 | rs34938615 | 14 22113999 | G | A | 0.0114 | -0.1220 | 0.0264 | 3.90E-06 |
|  | 14 | rs10138490 | 14 80883798 | C | T | 0.0605 | -0.0535 | 0.0114 | 2.80E-06 |
|  | 15 | rs7205927 | 16 48911591 | C | A | 0.4119 | -0.0258 | 0.0055 | 3.20E-06 |
|  | 16 | rs3745438 | 19 54972918 | C | T | 0.0337 | -0.0713 | 0.0156 | 4.90E-06 |
|  | 17 | rs67450584 | 22 44202370 | T | C | 0.1580 | 0.0367 | 0.0075 | 8.60E-07 |
| Folate (VB9) | 1 | rs78074774 | 2:34515489 | T | C | 0.0447 | 0.0601 | 0.0132 | 4.90E-06 |
|  | 2 | rs3772928 | 3:115406478 | T | C | 0.5754 | -0.0273 | 0.0055 | 8.20E-07 |
|  | 3 | rs76630415 | 7:14144445 | T | G | 0.212 | -0.0374 | 0.0067 | 2.40E-08 |
|  | 4 | rs2449166 | 8:3463535 | T | C | 0.4714 | 0.0252 | 0.0055 | 4.00E-06 |
|  | 5 | rs45442894 | 10:16870693 | A | C | 0.0141 | 0.1096 | 0.0240 | 5.00E-06 |
|  | 6 | rs7074988 | 10:87848038 | A | G | 0.0637 | -0.0513 | 0.0111 | 4.00E-06 |
|  | 7 | rs1502443 | 16:77060066 | G | C | 0.63 | 0.0259 | 0.0056 | 4.30E-06 |
|  | 8 | rs16956822 | 17:7499349 | G | A | 0.0262 | -0.0793 | 0.0172 | 3.90E-06 |
|  | 9 | rs148031795 | 18:482310 | T | C | 0.015 | 0.1044 | 0.0224 | 3.10E-06 |
|  | 10 | rs8085166 | 18:3016615 | G | A | 0.6769 | 0.0278 | 0.0058 | 1.70E-06 |
|  | 11 | rs79975477 | 20:13683165 | T | C | 0.031 | 0.0731 | 0.0156 | 2.80E-06 |
|  | 12 | rs76802001 | 22:31013399 | G | A | 0.0362 | -0.0677 | 0.0148 | 4.60E-06 |
|  | 13 | rs79748722 | 22:46147495 | C | T | 0.028 | -0.0757 | 0.0165 | 4.40E-06 |
| VB12 | 1 | rs2336573 | 19:8367709 | T | C | 0.031 | 0.32 | 0.021 | 1.10E-51 |
|  | 2 | rs1131603 | 22:31018975 | C | T | 0.055 | 0.19 | 0.017 | 4.30E-28 |
|  | 3 | rs3742801 | 14:74759006 | T | C | 0.294 | 0.045 | 0.008 | 5.30E-08 |
|  | 4 | rs2270655 | 4:146576418 | G | C | 0.941 | 0.066 | 0.016 | 3.50E-05 |
|  | 5 | rs12272669 | 11:71392610 | A | G | 0.0022 | 0.51 | 0.086 | 3.00E-09 |
|  | 6 | rs34324219 | 11:59623378 | C | A | 0.881 | 0.21 | 0.012 | 8.80E-71 |
|  | 7 | rs7788053 | 7:86773722 | A | G | 0.254 | 0.046 | 0.009 | 2.10E-07 |
|  | 8 | rs602662 | 19:49206985 | A | G | 0.596 | 0.16 | 0.008 | 4.10E-96 |
|  | 9 | rs1801222 | 10:17156151 | G | A | 0.593 | 0.11 | 0.007 | 1.10E-52 |
|  | 10 | rs41281112 | 13:100518634 | C | T | 0.948 | 0.17 | 0.016 | 9.60E-27 |
|  | 11 | rs1141321 | 6:49412433 | C | T | 0.627 | 0.061 | 0.007 | 1.40E-16 |
|  | 12 | rs5753231 | 22:31003069 | C | T | 0.79 | 0.064 | 0.01 | 7.50E-10 |
|  | 13 | rs56077122 | 10:17207015 | A | C | 0.335 | 0.087 | 0.009 | 4.80E-21 |
|  | 14 | rs34528912 | 11:59631535 | T | C | 0.036 | 0.17 | 0.021 | 2.10E-15 |
|  | 15 | rs117456053 | 11:59616831 | G | A | 0.976 | 0.16 | 0.027 | 1.90E-09 |

SNP, single nucleotide polymorphisms; SE, standard error; EAF: effect allele frequency; VB6: Vitamin B6; HCY: Homocysteine

**Supplementary Table S3.** Associations of genetic prediction of serum levels of folate, vitamin B6, vitamin B12 and homocysteine with sarcopenia-related traits in the MR analysis. (MR-Egger; Weighted median; Inverse variance weighted; Simple mode; Weighted mode; MR Egger (bootstrap). )

| Exposure-Outcome | SNP(n) | OR | 95%CI | *p* | low | hi |
| --- | --- | --- | --- | --- | --- | --- |
| Hcy — Low hand grip strength | 10 |  |  |  |  |  |
| MR Egger |  | 0.996 | 0.722-1.375 | 0.983 | 0.722 | 1.375 |
| Weighted median |  | 1.070 | 0.932-1.227 | 0.339 | 0.932 | 1.227 |
| Inverse variance weighted |  | 1.133 | 1.016-1.263 | **0.025** | 1.016 | 1.263 |
| Simple mode |  | 1.067 | 0.873-1.304 | 0.539 | 0.873 | 1.304 |
| Weighted mode |  | 1.065 | 0.910-1.247 | 0.451 | 0.910 | 1.247 |
| MR Egger (bootstrap) |  | 0.976 | 0.715-1.333 | 0.444 | 0.715 | 1.333 |
| Vit B_12_ — Low hand grip strength | 11 |  |  |  |  |  |
| MR Egger |  | 1.083 | 0.989-1.187 | 0.120 | 0.989 | 1.187 |
| Weighted median |  | 1.052 | 0.978-1.132 | 0.174 | 0.978 | 1.132 |
| Inverse variance weighted |  | 1.034 | 0.978-1.092 | 0.235 | 0.978 | 1.092 |
| Simple mode |  | 1.073 | 0.967-1.190 | 0.183 | 0.967 | 1.190 |
| Weighted mode |  | 1.055 | 0.982-1.134 | 0.181 | 0.982 | 1.134 |
| MR Egger (bootstrap) |  | 1.107 | 0.979-1.252 | **0.048** | 0.979 | 1.252 |
| Vit B_6_ — Low hand grip strength | 16 |  |  |  |  |  |
| MR Egger |  | 0.934 | 0.487-1.790 | 0.840 | 0.487 | 1.790 |
| Weighted median |  | 1.136 | 0.836-1.543 | 0.415 | 0.836 | 1.543 |
| Inverse variance weighted |  | 1.093 | 0.829-1.440 | 0.528 | 0.829 | 1.440 |
| Simple mode |  | 1.623 | 0.875-3.009 | 0.145 | 0.875 | 3.009 |
| Weighted mode |  | 1.520 | 0.887-2.606 | 0.149 | 0.887 | 2.606 |
| MR Egger (bootstrap) |  | 1.286 | 0.789-2.097 | 0.148 | 0.789 | 2.097 |
| Folate — Low hand grip strength | 13 |  |  |  |  |  |
| MR Egger |  | 1.281 | 0.626-2.618 | 0.512 | 0.626 | 2.618 |
| Weighted median |  | 0.809 | 0.581-1.128 | 0.211 | 0.581 | 1.128 |
| Inverse variance weighted |  | 1.012 | 0.741-1.384 | 0.938 | 0.741 | 1.384 |
| Simple mode |  | 0.703 | 0.386-1.280 | 0.272 | 0.386 | 1.280 |
| Weighted mode |  | 0.708 | 0.417-1.201 | 0.224 | 0.417 | 1.201 |
| MR Egger (bootstrap) |  | 0.732 | 0.406-1.319 | 0.146 | 0.406 | 1.319 |
| Exposure-Outcome | SNP(n) | β | 95%CI | *p-value* | low | hi |
| Hcy — ALM | 10 |  | | | | |
| MR Egger |  | 0.004 | -0.074, 0.082 | 0.920 | -0.074 | 0.082 |
| Weighted median |  | -0.051 | -0.086, -0.017 | **0.005** | -0.086 | -0.017 |
| Inverse variance weighted |  | -0.043 | -0.069, -0.016 | **0.001** | -0.069 | -0.016 |
| Simple mode |  | -0.055 | -0.107, -0.002 | 0.080 | -0.017 | -0.002 |
| Weighted mode |  | -0.051 | -0.095, -0.007 | 0.054 | -0.095 | -0.007 |
| MR Egger (bootstrap) |  | -0.049 | -0.128, -0.031 | 0.103 | -0.128 | -0.031 |
| Vit B_12_ — ALM | 11 |  | | | | |
| MR Egger |  | -0.019 | -0.060, 0.022 | 0.387 | -0.060 | 0.022 |
| Weighted median |  | -0.016 | -0.035, 0.002 | 0.085 | -0.035 | 0.002 |
| Inverse variance weighted |  | -0.010 | -0.035, 0.015 | 0.436 | -0.035 | 0.015 |
| Simple mode |  | -0.025 | -0.068, 0.019 | 0.297 | -0.068 | 0.019 |
| Weighted mode |  | -0.019 | -0.042, 0.003 | 0.106 | -0.042 | 0.003 |
| MR Egger (bootstrap) |  | -0.038 | -0.064, 0.003 | **0.002** | -0.064 | 0.003 |
| Vit B_6_ — ALM | 17 |  | | | | |
| MR Egger |  | 0.045 | -0.057, 0.146 | 0.402 | -0.057 | 0.146 |
| Weighted median |  | -0.018 | -0.082, 0.045 | 0.561 | -0.082 | 0.045 |
| Inverse variance weighted |  | -0.013 | -0.059, 0.033 | 0.575 | -0.059 | 0.033 |
| Simple mode |  | -0.016 | -0.133, 0.101 | 0.792 | -0.133 | 0.101 |
| Weighted mode |  | -0.024 | -0.135, 0.086 | 0.679 | -0.135 | 0.086 |
| MR Egger (bootstrap) |  | -0.063 | -0.170, 0.045 | 0.118 | -0.170 | 0.045 |
| Folate — ALM | 13 |  | | | | |
| MR Egger |  | -0.007 | -0.128, 0.113 | 0.902 | -0.128 | 0.113 |
| Weighted median |  | 0.020 | -0.047, 0.087 | 0.575 | -0.047 | 0.087 |
| Inverse variance weighted |  | 0.018 | -0.036, 0.072 | 0.507 | -0.036 | 0.072 |
| Simple mode |  | 0.022 | -0.090, 0.134 | 0.700 | -0.090 | 0.134 |
| Weighted mode |  | 0.021 | -0.093, 0.136 | 0.736 | -0.093 | 0.136 |
| MR Egger (bootstrap) |  | 0.052 | -0.082, 0.187 | 0.215 | -0.082 | 0.187 |
|  | | | | | | |
| Hcy — Walking pace | 10 |  | | | | |
| MR Egger |  | 0.009 | -0.073, 0.092 | 0.833 | -0.073 | 0.092 |
| Weighted median |  | 0.005 | -0.016, 0.027 | 0.641 | -0.016 | 0.027 |
| Inverse variance weighted |  | 0.002 | -0.025, 0.028 | 0.895 | -0.025 | 0.028 |
| Simple mode |  | -0.001 | -0.034, 0.032 | 0.963 | -0.034 | 0.032 |
| Weighted mode |  | 0.003 | -0.026, 0.032 | 0.852 | -0.026 | 0.032 |
| MR Egger (bootstrap) |  | 0.052 | -0.004, 0.104 | **0.008** | -0.004 | 0.104 |
| Vit B_12_ — Walking pace | 9 |  | | | | |
| MR Egger |  | -0.007 | -0.031, 0.017 | 0.584 | -0.031 | 0.017 |
| Weighted median |  | -0.001 | -0.014, 0.012 | 0.847 | -0.014 | 0.012 |
| Inverse variance weighted |  | 0.002 | -0.010, 0.013 | 0.795 | -0.010 | 0.013 |
| Simple mode |  | -0.011 | -0.032, 0.010 | 0.333 | -0.032 | 0.010 |
| Weighted mode |  | -0.011 | -0.035, 0.013 | 0.442 | -0.035 | 0.013 |
| MR Egger (bootstrap) |  | 0.024 | 0.006, 0.042 | **0.002** | 0.006 | 0.042 |
| Vit B_6_ — Walking pace | 17 |  | | | | |
| MR Egger |  | 0.001 | -0.085, 0.087 | 0.983 | -0.085 | 0.087 |
| Weighted median |  | 0.037 | 0.001, 0.073 | **0.038** | 0.001 | 0.073 |
| Inverse variance weighted |  | 0.001 | -0.036, 0.039 | 0.944 | -0.036 | 0.039 |
| Simple mode |  | 0.040 | -0.025, 0.104 | 0.250 | -0.025 | 0.104 |
| Weighted mode |  | 0.040 | -0.019, 0.098 | 0.220 | -0.019 | 0.098 |
| MR Egger (bootstrap) |  | 0.023 | -0.037, 0.083 | 0.200 | -0.037 | 0.083 |
| Folate — Walking pace | 13 |  | | | | |
| MR Egger |  | 0.014 | -0.053, 0.080 | 0.699 | -0.053 | 0.080 |
| Weighted median |  | 0.021 | -0.014, 0.057 | 0.240 | -0.014 | 0.057 |
| Inverse variance weighted |  | 0.010 | -0.018, 0.039 | 0.481 | -0.018 | 0.039 |
| Simple mode |  | 0.022 | -0.035, 0.079 | 0.471 | -0.035 | 0.079 |
| Weighted mode |  | 0.023 | -0.034, 0.080 | 0.445 | -0.034 | 0.080 |
| MR Egger (bootstrap) |  | 0.001 | -0.075, 0.075 | 0.478 | -0.075 | 0.075 |

*p*＜0.05

**Supplementary Table S4.** Heterogeneity of MR analysis for serum levels of folate, vitamin B6, vitamin B12, homocysteine and sarcopenia-related traits.

| Exposure | Outcome | Method | heterogeneity test | | |
| --- | --- | --- | --- | --- | --- |
|  |  |  | Q | Q_df | Q_*p value* |
| HCY | Low hand grip strength | MR-Egger | 7.108 | 8 | 0.525 |
|  |  | Inverse variance weighted | 7.796 | 9 | 0.555 |
|  | ALM | MR-Egger | 6.794 | 8 | 0.559 |
|  |  | Inverse variance weighted | 8.351 | 9 | 0.499 |
|  | Walking pace | MR-Egger | 36.035 | 8 | **0.001** |
|  |  | Inverse variance weighted | 36.192 | 9 | **0.001** |
| VB6 | Low hand grip strength | MR-Egger | 28.261 | 14 | **0.013** |
|  |  | Inverse variance weighted | 28.817 | 15 | **0.017** |
|  | ALM | MR-Egger | 11.818 | 15 | 0.693 |
|  |  | Inverse variance weighted | 13.394 | 16 | 0.644 |
|  | Walking pace | MR-Egger | 42.970 | 15 | **0.001** |
|  |  | Inverse variance weighted | 42.970 | 16 | **0.001** |
| Folate | Low hand grip strength | MR-Egger | 22.434 | 11 | **0.021** |
|  |  | Inverse variance weighted | 23.490 | 12 | **0.024** |
|  | ALM | MR-Egger | 3.528 | 11 | 0.982 |
|  |  | Inverse variance weighted | 3.751 | 12 | 0.988 |
|  | Walking pace | MR-Egger | 13.662 | 11 | 0.252 |
|  |  | Inverse variance weighted | 13.676 | 12 | 0.322 |
| VB12 | Low hand grip strength | MR-Egger | 6.573 | 9 | 0.681 |
|  |  | Inverse variance weighted | 8.144 | 10 | 0.615 |
|  | ALM | MR-Egger | 38.860 | 9 | **0.001** |
|  |  | Inverse variance weighted | 40.23 | 10 | **0.001** |
|  | Walking pace | MR-Egger | 12.917 | 7 | 0.074 |
|  |  | Inverse variance weighted | 14.076 | 8 | 0.080 |

**Supplementary Table S5.** Effect sizes for Hcy, VB6 and Walking pace were estimated using a random effects model

| Exposure | Outcome | Method | SNP(n) | b | se | *p* |
| --- | --- | --- | --- | --- | --- | --- |
| Hcy | Walking pace | multiplicative random effects | 10 | 0.002 | 0.013 | 0.895 |
| VB6 | Walking pace | multiplicative random effects | 17 | 0.001 | 0.019 | 0.944 |

**Supplementary Table S6.** Associations of genetic prediction of serum levels of folate, vitamin B6, vitamin B12 and homocysteine with sarcopenia-related traits in the MR-Level pleiotropy analysis.

| Exposure | Outcome | Level pleiotropy | | |
| --- | --- | --- | --- | --- |
|  |  | Egger_intercept | SE | *p* |
| HCY | Low hand grip strength | 0.009 | 0.011 | 0.431 |
|  | ALM | -0.003 | 0.003 | 0.247 |
|  | Walking pace | -0.001 | 0.003 | 0.856 |
| VB6 | Low hand grip strength | 0.007 | 0.013 | 0.608 |
|  | ALM | -0.003 | 0.002 | 0.229 |
|  | Walking pace | 0.001 | 0.002 | 0.993 |
| Folate | Low hand grip strength | -0.010 | 0.015 | 0.487 |
|  | ALM | 0.001 | 0.002 | 0.646 |
|  | Walking pace | -0.001 | 0.001 | 0.917 |
| VB12 | Low hand grip strength | -0.009 | 0.007 | 0.242 |
|  | ALM | 0.002 | 0.003 | 0.587 |
|  | Walking pace | 0.001 | 0.002 | 0.454 |

**Supplementary Table S7.** Comparison Table of Baseline Data of Different Homocysteine Trajectory Groups of 1322 Physical Examination Participants.

| Variable | Low-stable group（n=1137） | Medium-stable group（n=77） | High-stable group（n=108） | *X^2^*/*F* | *p* |
| --- | --- | --- | --- | --- | --- |
| Age (years, Mean±SD) | 36.03±7.70 | 40.82±11.91 | 37.94±11.45 | 13.657 | <0.001 |
| Sex [n (%)] |  |  |  | 60.730 | <0.001 |
| Male | 630(55.4) | 23(29.9) | 23(21.3) |  |  |
| Female | 507(44.6) | 54(70.1) | 85(78.7) |  |  |
| Physical Activity [n (%)] |  |  |  | 22.053 | ＜0.001 |
| None/Sedentary | 225(19.8) | 30(39.0) | 31(28.7) |  |  |
| Occasional | 622(54.7) | 33(42.9) | 45(41.7) |  |  |
| Regular | 290(25.5) | 14(18.2) | 32(29.6) |  |  |
| Smoking Status [n (%)] |  |  |  | 107.982 | ＜0.001 |
| Never | 537(47.2) | 28(36.4) | 38(35.2) |  |  |
| Occasional | 391(34.4) | 23(29.9) | 33(30.6) |  |  |
| Current | 198(17.4) | 12(15.6) | 28(25.9) |  |  |
| Former | 11(1.0) | 14(18.2) | 9(8.3) |  |  |
| Alcohol Consumption [n (%)] |  |  |  | 47.377 | ＜0.001 |
| Never | 256(22.5) | 41(53.2) | 42(38.9) |  |  |
| Occasional | 282(24.8) | 12(15.6) | 18(16.7) |  |  |
| Current | 493(43.4) | 20(26.0) | 38(35.2) |  |  |
| Former | 106(9.3) | 4(5.2) | 10(9.3) |  |  |
| BMI, (kg/m², Mean ± SD) | 24.35±3.17 | 23.59±3.13 | 23.28±3.06 | 7.212 | 0.001 |
| SBP (mmHg, Mean ± SD) | 119.88±15.30 | 116.55±13.77 | 121.65±18.29 | 2.436 | 0.088 |
| DBP (mmHg, Mean ± SD) | 75.25±9.79 | 72.40±9.59 | 74.67±11.55 | 2.972 | 0.052 |
| UA (μmol/L, Mean ± SD) | 336.49±90.31 | 316.53±100.60 | 283.44±80.82 | 18.108 | <0.001 |
| FBG (mmol/L, Mean ± SD) | 5.44±1.43 | 5.51±1.77 | 5.02±0.63 | 4.773 | 0.009 |
| TC (mmol/L, Mean ± SD) | 5.01±1.03 | 4.90±1.02 | 4.80±1.03 | 2.276 | 0.103 |
| TG (mmol/L, Mean ± SD) | 1.75±1.12 | 1.39±0.83 | 1.41±0.92 | 8.165 | <0.001 |
| LDL-C（mmol/L, Mean ± SD） | 3.26±0.90 | 3.06±0.69 | 3.01±0.71 | 5.300 | 0.005 |
| HDL-C（mmol/L, Mean ± SD） | 1.33±0.28 | 1.27±0.33 | 1.02±0.27 | 58.355 | <0.001 |
| Sarcopenia [n (%)] | 76(6.7) | 15(19.5) | 27(25.0) | 51.907 | <0.001 |

**
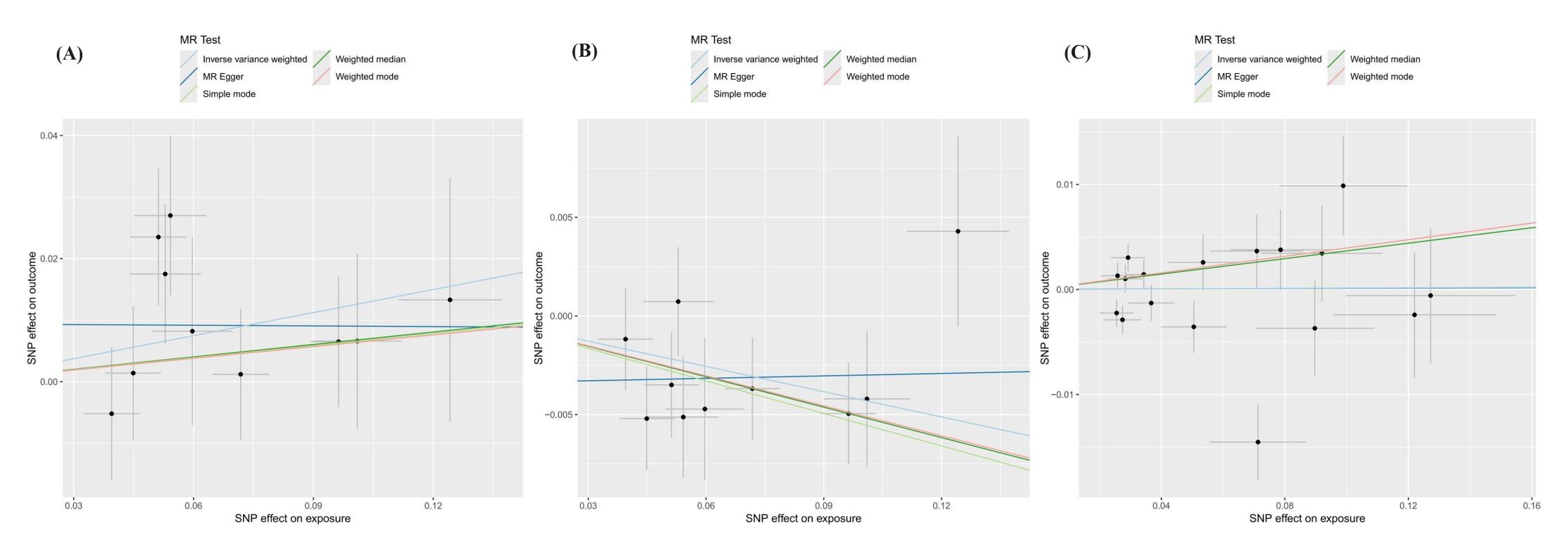
**

**Supplementary Figure S1.** Scatter plot of statistically significant results with IVW or default method as the primary method. (A) homocysteine and hand grip strength. (B) homocysteine and ALM. (C) VB6 and Walking pace. Five lines show the estimated effect sizes by the five MR Default methods (inverse-variance weighted, MR-Egger, simple mode, weighted mode and weighted median)


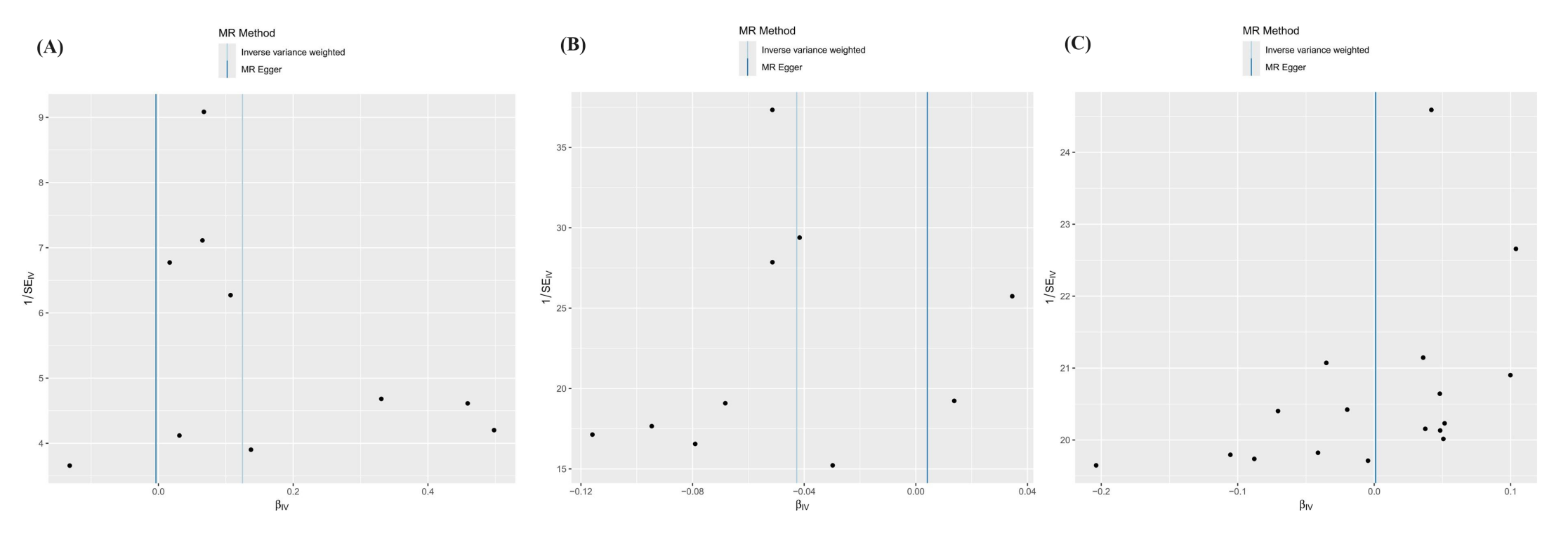


**Supplementary Figure S2.** Funnel plot of statistically significant results with IVW or default method as the primary method. (A) homocysteine and hand grip strength. (B) homocysteine and ALM. (C) VB6 and Walking pace. In the graphical visualization, the boundary line with IVW as the main method is centered, and three graphs are symmetric, which suggests that there is no heterogeneity in our results. Using the MR-Egger method as a boundary line, Figures 2A and 2B may exhibit asymmetry, which can be attributed to the limited number of SNP and the limitations of graphical visualization. Despite such problems, combined with more rigorous quantitative sensitivity analyses, including F-statistics, MR-Egger intercepts, we did not find significant results for heterogeneity.


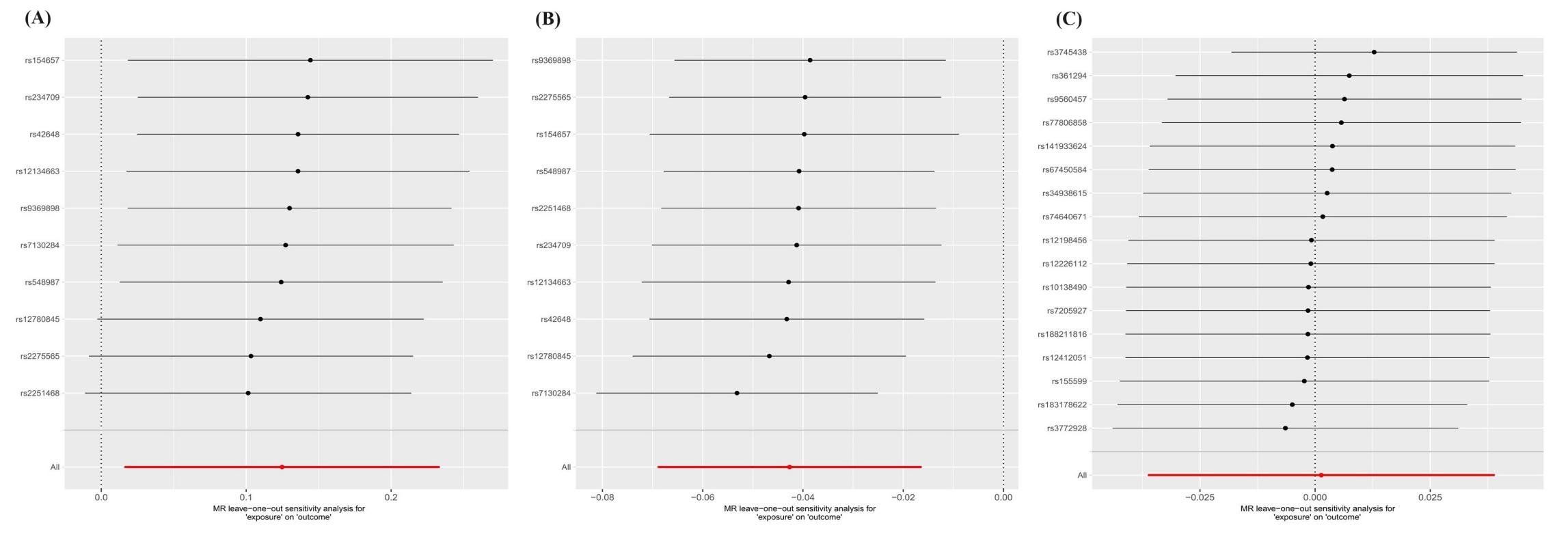


**Supplementary Figure S3.** Leave-one-out plots of statistically significant results with IVW or default method as the primary method. (A) homocysteine and hand grip strength. (B) homocysteine and ALM. (C) VB6 and Walking pace. We used the leave-one-out method to examine the analysis results of the remaining SNPS after removing each SNP one by one. The summary results basically showed negligible fluctuations, which confirmed the stability and reliability of the results. However, Figure 3C shows that there is a clear sensitivity, and we re-estimated the effect size of VB6 and Walking pace using a random effects model for the existing problems (**Table S5**). The results of the random effects model (*p*=0.944) refute the conclusion of a causal relationship between VB6 and working pace in the Weighted median approach.

**
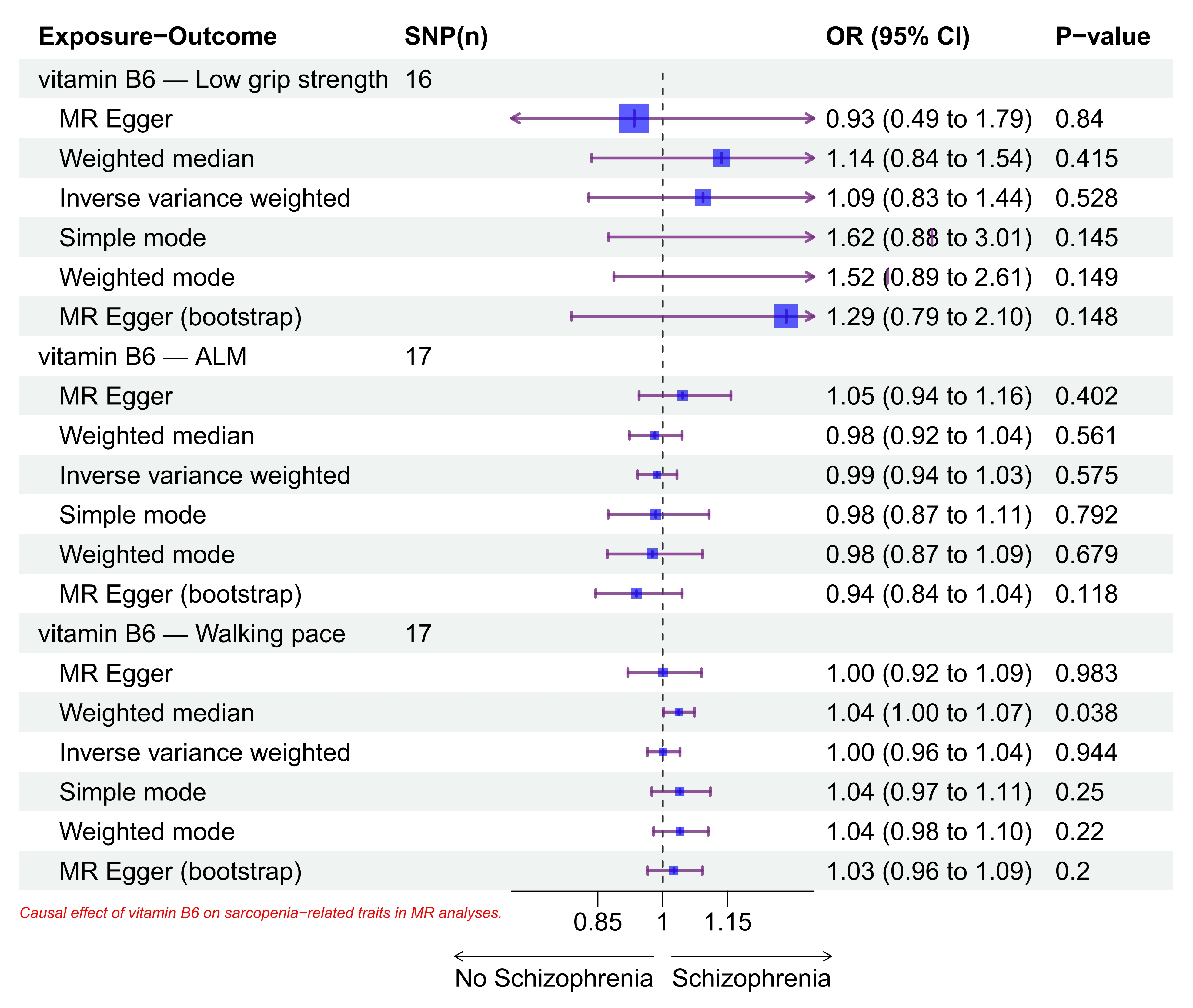
**

**Supplementary Figure S4.** Causal effect of VB6 on sarcopenia-related traits in MR analyses.

**
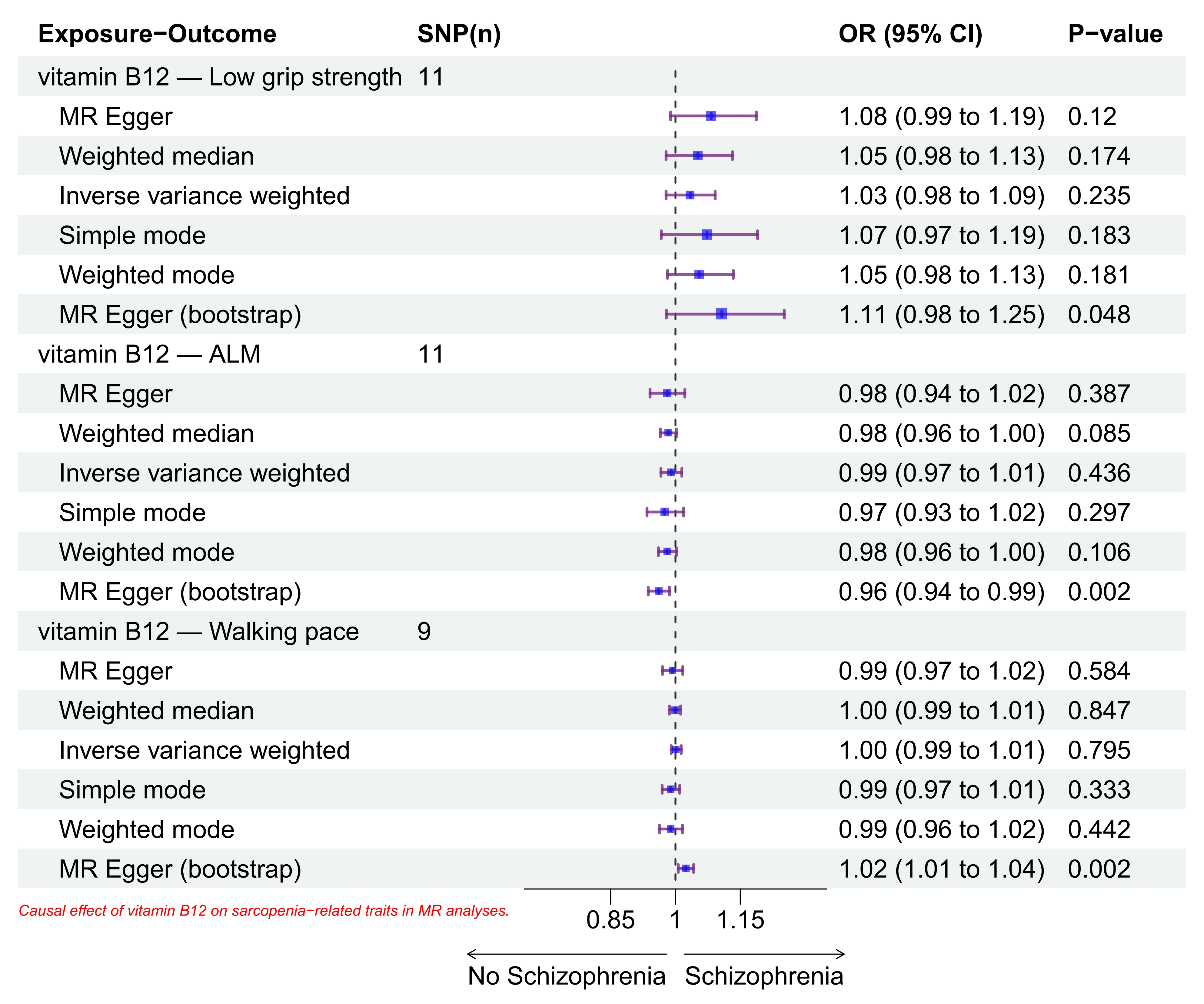
**

**Supplementary Figure S5. Causal effect of VB12 on sarcopenia-related traits in MR analyses.**
